# AR-regulated ZIC5 contributes to the aggressiveness of prostate cancer

**DOI:** 10.1038/s41420-022-01181-4

**Published:** 2022-09-20

**Authors:** Yi-Fan Tan, Yang Zhang, Sheng-Yang Ge, Fan Zhong, Chuan-Yu Sun, Guo-Wei Xia

**Affiliations:** 1 Department of Urology, Huashan Hospital, Fudan University, Shanghai, 200040 China; 2 Department of Systems Biology for Medicine, and Institutes of Biomedical Sciences, Shanghai Medical College, Fudan University, Shanghai, 200032 China

**Keywords:** Prostate cancer, Cell migration

## Abstract

The mechanisms by which prostate cancer (PCa) progresses to the aggressive castration-resistant stage remain uncertain. Zinc finger of the cerebellum 5 (ZIC5), a transcription factor belonging to the ZIC family, is involved in the pathology of various cancers. However, the potential effect of ZIC5 on PCa malignant progression has not been fully defined. Here, we show that ZIC5 is upregulated in PCa, particularly in metastatic lesions, in positive association with poor prognosis. Genetic inhibition of ZIC5 in PCa cells obviously attenuated invasion and metastasis and blunted the oncogenic properties of colony formation. Mechanistically, ZIC5 functioned as a transcription factor to promote TWIST1-mediated EMT progression or as a cofactor to strengthen the β-catenin-TCF4 association and stimulate Wnt/β-catenin signaling. Importantly, ZIC5 and the androgen receptor (AR) form a positive feed-forward loop to mutually stimulate each other’s expression. AR, in cooperation with its steroid receptor coactivator 3 (SRC-3), increased ZIC5 expression through binding to the miR-27b-3p promoter and repressing miR-27b-3p transcription. In turn, ZIC5 potentiated AR, AR-V7, and AR targets’ expression. Besides, ZIC5 inhibition reduced AR and AR-V7 protein expression and enhanced the sensitivity of PCa to enzalutamide (Enz) treatment, both in vitro and in vivo. These findings indicate that the reciprocal activation between AR and ZIC5 promotes metastasis and Enz resistance of PCa and suggest the therapeutic value of cotargeting ZIC5 and AR for the treatment of advanced PCa.

## Introduction

Prostate cancer (PCa) is the most common urological tumor among men in developed countries and constitutes therefore a global health problem [1]. With worldwide estimations of approximately 1.4 million new cases and more than 370,000 deaths in 2020, PCa ranks as the second most commonly diagnosed malignancy and the fifth leading cause of cancer-related mortality in males [2]. Early stage (T1-T2) tumors confined to the prostate organ can be cured by radical surgery or radiotherapy, leading to significantly improved survival rates [3]. Nevertheless, more than 30% of patients who undergo radical treatment experience local recurrence or even develop distant metastases, resulting in the disease progressing to incurable, advanced stages [4, 5]. Androgen receptor (AR) signaling drives PCa cell proliferation and progression and is thus the main target of most drugs employed for PCa therapy [6, 7]. AR functions as a transcription factor that binds to DNA promoter regions to modulate the expression of numerous genes implicated in the control of cell cycle and cell differentiation [8]. However, the effect of AR signaling in PCa metastasis remains incompletely characterized. Recent discoveries suggested that AR retains a dominant role and acts as a stimulus to support tumor metastasis [9–12]. Furthermore, aberrant reactivation of AR signaling is an essential factor in the development of endocrine therapy resistance [13, 14]. Hence, a detailed understanding of PCa biology and elucidation of the mechanisms underlying AR-driven tumor aggressiveness will help develop novel treatments for patients with advanced PCa.

The zinc finger of the cerebellum (ZIC) gene family consists of five members (ZIC1–5) originally identified in the adult mice cerebellum [15]. ZIC proteins act as classical zinc finger-containing transcriptional regulators and play a critical role in neural, skeletal, and muscle development [16]. Indeed, aberrant expression of ZIC-encoding genes is associated with a series of congenital disorders, including spina bifida, skeletal abnormalities, and exencephaly [17, 18]. ZIC5 is located on human chromosome 13 and encodes the ZIC5 protein, which contains five tandemly repeated, highly conserved Cys2His2-type zinc finger domains [18]. ZIC5 modulates the expression of target genes by acting as a transcriptional activator, repressor, or cofactor [17]. The ZIC5 gene is normally highly expressed in the cerebellum, and its deficiency induces neural crest abnormalities and neural tube defects in vivo [18]. Besides its important role in organ development, studies in recent years suggested that ZIC5 is involved in the pathology of various diseases, including cancer, and highlighted also some potential mechanisms [19–21]. For example, it was found that characteristic high ZIC5 expression promotes progression and metastasis of lung and hepatocellular carcinoma through mechanisms involving, respectively, enhanced CCNB1/CDK1 complex expression and Wnt/β-catenin signaling stimulation [22, 23]. Another study revealed that ZIC5 stimulated colorectal cancer cell proliferation via the CDK1/CDC25c pathway, and depletion of ZIC5 exerted antineoplastic effects [24]. In turn, ZIC5 inhibition enhanced transcriptional elongation of CDH1 (E-cadherin), whereas ZIC5 overexpression triggered PDGFD-mediated activation of FAK and STAT3 signaling, thereby promoting melanoma aggressiveness [25].

Despite mounting evidence of the pervasive influence of ZIC5 in multiple cancer types, the association between ZIC5 and AR signaling or with PCa tumor aggressiveness has not been fully defined. In the present study, we conducted bioinformatics analysis on human PCa databases, as well as a series of in vitro and in vivo experiments ascertaining function and regulatory and effector mechanisms of ZIC5 in PCa. Our results reveal a novel positive feed-forward loop between AR and ZIC5 as a potentially major contributor to malignant progression in PCa and suggest the therapeutic value of ZIC5 inhibition.

## Results

### ZIC5 is overexpressed in human PCa specimens and cell lines

**Fig. 1:**
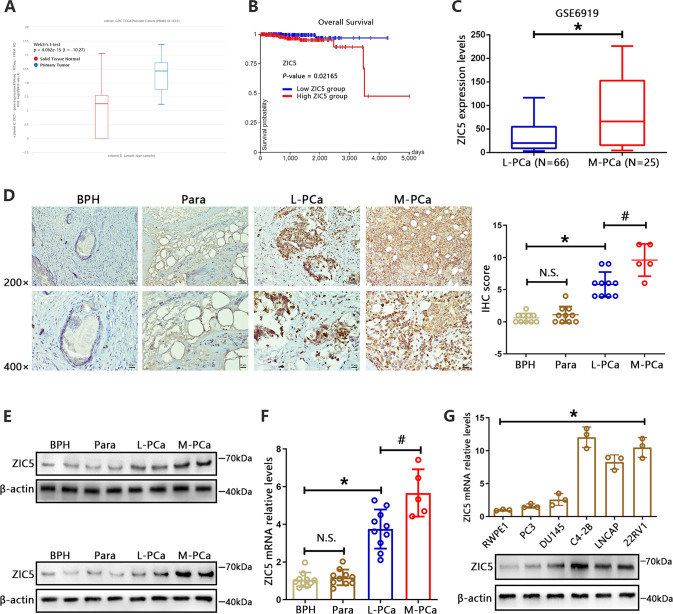
ZIC5 is overexpressed in human PCa tissues and cell lines. **A** ZIC5 expression in human cancerous and normal prostate tissues in TCGA database from UCSC Xena platform. **B** Kaplan–Meier survival analysis based on ZIC5 expression in PCa patients. **C** Expression of ZIC5 in localized tumor and metastatic PCa (M-PCa) patient samples (GEO dataset GSE6919). *P < 0.05 vs. localized PCa (L-PCa) tumors. **D** IHC analysis of ZIC5 expression in BPH, localized PCa and adjacent normal tissue, and metastatic PCa specimens. Corresponding IHC scores were obtained according to the percentage of positive cells and the intensity of staining. N.S. (nonsignificant), *P* > 0.05 vs. BPH, *P < 0.05 vs. BPH, ^#^*P* < 0.05 vs. localized PCa tumors. **E** Western blot images depicting ZIC5 expression in clinical tissues. **F** RT-qPCR analysis of relative ZIC5 mRNA levels in clinical tissues. N.S., *P* > 0.05 compared to BPH. *P < 0.05, compared to BPH. ^**#**^*P* < 0.05, compared to L-PCa. **G** Analysis of relative ZIC5 expression levels in normal prostate epithelial RWPE1 cells and PCa cell lines by RT-qPCR and western blot assays. Data are presented as means ± SD (*n* = 3). *P < 0.05 vs. RWPE1 data.

To explore the potential impact of ZIC5 on PCa, we first extracted gene expression profiles from TCGA using the UCSC Xena platform. As shown in Fig. 1A, ZIC5 expression was markedly elevated in PCa tissues compared with normal ones. To investigate the prognostic value of ZIC5 in PCa, we performed survival analysis and log-rank tests on the above TCGA-PCa dataset. Results showed that higher expression of ZIC5 correlated with worse overall survival in PCa patients (Fig. 1B). A previous study reported that ZIC5 overexpression promotes melanoma aggressiveness and metastatic spread [25]. However, whether ZIC5 overexpression contributes to tumor metastasis in PCa remains unclear. Thus, PCa GEO datasets were included in our analysis. Based on data from GSE6919, we found that the expression levels of ZIC5 were notably higher in metastatic PCa than in localized carcinomas (Fig. 1C). In addition, analysis of the GSE3325 dataset also revealed the same trend (Supplementary Fig. S1C). To further validate these findings, four sets of clinical samples were collected, including benign prostatic hyperplasia (BPH) tissue samples, localized PCa and adjacent non-tumor samples, and metastatic PCa tumor samples. Immunohistochemical staining showed that the expression of ZIC5 was predominately located in the nucleus, and the number of ZIC5-positive cells increased along with disease aggressiveness. In particular, the most intense ZIC5 staining was found in metastatic tumor tissues (Fig. 1D). Furthermore, reinforcing a potential contribution of ZIC5 to PCa metastasis, RT-qPCR and western blot data indicated that ZIC5 levels were obviously higher in metastatic lesions than in localized tumors (Fig. 1E, F).

Next, we assessed ZIC5 expression levels in five PCa cell lines (PC3, DU145, C4-2B, LNCAP, and 22RV1) and in normal human prostate epithelial RWPE1 cells. Similar to results from human PCa tissues, ZIC5 expression was significantly upregulated in the PCa cell lines compared to RWPE1 cells (Fig. 1G). These results indicated that overexpression of ZIC5 correlates with poor prognosis in PCa patients and is probably involved in the progression and metastasis of PCa.

### ZIC5 promotes EMT progression in PCa cell lines

We further explored the biological function of ZIC5 in PCa cells. Because C4-2B and 22RV1 cells exhibited higher ZIC5 expression levels than the other three PCa cell lines examined (Fig. 1G), those two cell lines were selected for subsequent analyses. We then applied RNA silencing to suppress ZIC5 expression and used a lentiviral plasmid to overexpress ZIC5 in both C4-2B and 22RV1 cells. High transfection efficiency was confirmed by RT-qPCR and western blotting assays (Supplementary Fig. 1A, B). Then, wound healing, Transwell-Matrigel, and colony formation assays were performed to measure the migration and invasion abilities and colony formation capacities of C4-2B and 22RV1 cells. Knockdown of ZIC5 expression markedly attenuated migration and invasion and colony formation potential, while forced ZIC5 expression conferred stronger migratory, invasive, and colony formation abilities in both cell lines (Supplementary Fig. 2A, B and Supplementary Fig. 1D, E). In addition, we also evaluated the effect of ZIC5 on the metastasis in PC3 cells, an AR-negative PCa cell line that is widely used in prostate cancer research. The results showed that ZIC5 inhibition could barely affect PC3 cell invasion and migration, whereas restoration of ZIC5 slightly induced metastasis of PC3 cells (Supplementary Fig. 1F, G), which might be due to the moderate expression of ZIC5 and AR in PC3 cells.

**Fig. 2:**
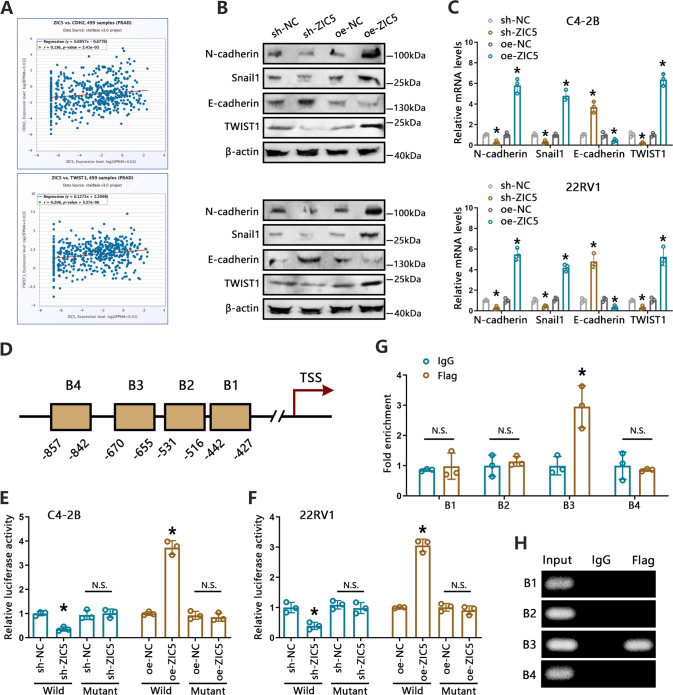
ZIC5 regulates EMT progression in PCa cell lines. **A** Analysis of the correlation between ZIC5, CDH2, and TWIST1 expression in PCa patients (TCGA-PCa data from ENCORI). **B**, **C** Analysis of N-cadherin, Snail1, E-cadherin, and TWIST1 expression in C4-2B and 22RV1 cells transfected with ZIC5-targeted shRNA (sh-ZIC5) or ZIC5 overexpression plasmid (oe-ZIC5), measured by western blotting (**B**) or RT-qPCR (**C**). *P < 0.05, relative to sh-NC or oe-NC. **D** Putative ZIC5-binding sites on the TWIST1 promoter region. **E**, **F** ZIC5**-**targeted shRNA or ZIC5 overexpression plasmid and TWIST1 promoter-driven wild-type luciferase reporter or mutant vectors were cotransfected into C4-2B (**E**) and 22RV1 (**F**) cells. Luciferase assays were performed to examine ZIC5/TWIST1 interaction. *P < 0.05, relative to sh-NC or oe-NC. **G** ChIP-qPCR analysis of ZIC5 binding to the promoter region of TWIST1. C4-2B cells were transfected with Flag-tagged ZIC5 or control vectors, followed by immunoprecipitation with anti-Flag or anti-IgG antibodies. Purified IgG was used as control. Experiments were performed in triplicate. *P < 0.05 vs. IgG. **H** Representative ChIP results showing ZIC5 recruitment onto the TWIST1 promoter.

Given that EMT is a major step in the process of cancer cell metastasis [26], and ZIC5 was reported to modulate the expression of EMT genes [25]. we investigated whether ZIC5 promotes EMT in C4-2B and 22RV1 cells. Analysis of the association between ZIC5 and EMT-related markers in TCGA-PCa patient data using the ENCORI platform revealed that ZIC5 expression correlated positively with TWIST1 and CDH2 (N-cadherin) expression in PCa specimens (Fig. 2A). Furthermore, after ZIC5 silencing, both RT-qPCR and western blotting showed significantly increased levels of E-cadherin, a protein responsible for epithelial adherens junction formation, and a remarkable decline in the levels of mesenchymal-associated proteins, namely N-cadherin, TWIST1, and Snail1. In contrast, exogenous expression of ZIC5 upregulated the expression of EMT markers in both C4-2B and 22RV1 cells (Fig. 2B, C).

Next, we explored the potential mechanisms of how ZIC5 is involved in the progression of EMT in C4-2B and 22RV1 cells. Based on the above findings, we conducted studies to verify transcriptional activation of TWIST1, a critical activator of the EMT process [27], by ZIC5. Bioinformatics prediction was carried out and identified four potential ZIC5-binding sites on the promoter region of TWIST1 (Fig. 2D). We then applied luciferase reporter assays to determine whether ZIC5 directly binds to the TWIST1 gene promoter. Results revealed that ZIC5 silencing reduced, whereas its overexpression drastically increased, the activity of the wild-type TWIST1 promoter in both C4-2B and 22RV1 cells. In contrast, neither silencing nor upregulation of ZIC5 altered the activity of a mutant TWIST1 promoter construct (Fig. 2E, F). Subsequently, we verified the interaction between ZIC5 and the TWIST1 promoter through ChIP assays. Four potential binding sites, namely B1 (−427 to −442 bp), B2 (−516 to −531 bp), B3 (−655 to −670 bp) and B4 (−842 to −857 bp), were included in our study. A strong enhancement in the recruitment of ZIC5 was found only on the B3 region of the TWIST1 promoter, indicating that ZIC5 binds to a region located 655 to 670 bp upstream of the transcription start site (TSS) (Fig. 2G, H). Next, to determine whether TWIST1 expression mediates ZIC5-induced motility and metastasis of PCa cells, siRNA-mediated TWIST1 silencing was induced in C4-2B and 22RV1 cells. Wound healing and Transwell-Matrigel assays showed that depletion of TWIST1 significantly restricted ZIC5-induced migration and invasion of PCa cells (Supplementary Fig. 2C, D). Collectively, our data proved that ZIC5 promotes EMT via enhancing TWIST1 transcription, thus facilitating metastasis of PCa cells.

### ZIC5 regulates Wnt/β-catenin signaling in vitro

**Fig. 3:**
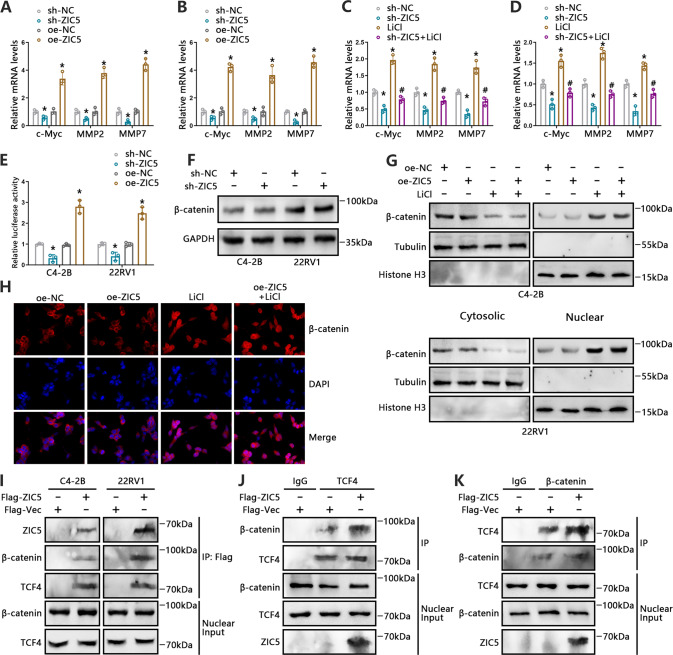
ZIC5 increases Wnt/β-catenin signaling by potentiating the interaction between β-catenin and TCF4. **A**, **B** C4-2B (**A**) and 22RV1 (**B**) cells were transfected with sh-ZIC5 or oe-ZIC5. Relative expression of Wnt/β-catenin target genes (c-Myc, MMP2, and MMP7) was measured by RT-qPCR. *P < 0.05, relative to sh-NC or oe-NC. **C**, **D** C4-2B (**C**) and 22RV1 (**D**) cells transfected with sh-NC or sh-ZIC5 and treated with LiCl (20 mmol/L) for 24 h. Relative expression levels of c-Myc, MMP2 and MMP7 were analyzed by RT-qPCR. *P < 0.05, vs. sh-NC, ^#^*P* < 0.05 vs. sh-ZIC5. **E** TCF/LEF Luciferase reporter assay results depicting Wnt/β-catenin signaling activity in C4-2B and 22RV1 cells after ZIC5 knockdown or overexpression. *P < 0.05 vs. sh-NC or oe-NC. **F** Western blot analysis of overall β-catenin expression in C4-2B and 22RV1 cells transfected with sh-NC or sh-ZIC5. **G** Western blot assessment of β-catenin protein levels in the cytoplasm and nucleus of C4-2B and 22RV1 cells treated as indicated. **H** The cellular distribution of β-catenin assessed through immunofluorescence staining. Scale bars, 20 μm. **I** Co-IP analysis of interactions between ZIC5 and β-catenin/TCF4 in C4-2B and 22RV1 cells transfected with Flag-tagged ZIC5 or control vector. **J**, **K** Flag-tagged ZIC5 or control vectors were transfected into 293T cells and Co-IP was performed on nuclear extracts probed with anti-TCF4 (**J**) or anti-β-catenin (**K**) antibodies.

Aberrant activation of the Wnt/β-catenin pathway is closely associated with cancer progression and metastasis [28]. GEPIA analyses showed a potential link between ZIC5 and Wnt/β-catenin signaling genes, namely CTNNB1 (β-catenin) and GSK3B (GSK-3β), in the TCGA-PCa dataset (Supplementary Fig. 3A). We therefore surmised that ZIC5 might regulate the Wnt/β-catenin pathway to support PCa metastasis. Indeed, compared to control cells, a markedly increased expression of Wnt/β-catenin downstream genes, including c-Myc, MMP2, and MMP7, was noted in ZIC5-overexpressing PCa cells (Fig. 3A, B). In contrast, ZIC5 silencing was associated with significant repression of the above genes (Fig. 3A, B). Of note, the former effect could be blunted by application of LiCl, which enhances β-catenin activity by inhibiting GSK-3β (Fig. 3C, D). In addition, stimulation of Wnt/β-catenin signaling via LiCl markedly abrogated the inhibitory effect of ZIC5 knockdown on migration and invasion of C4-2B and 22RV1 cells (Supplementary Fig. 3B, C). These data suggest that ZIC5 promotes PCa cell metastasis through Wnt/β-catenin pathway activation.

To assess the above hypothesis, the effect of ZIC5 silencing and overexpression on Wnt/β-catenin activation was examined using a TCF/LEF luciferase reporter assay. Supporting our assumptions, ZIC5 knockdown drastically reduced, while ZIC5 overexpression significantly augmented, the luciferase activity of the TCF/LEF-responsive reporter in PCa cells (Fig. 3E). Since the translocation of β-catenin into the nucleus is a critical step in the transduction of WNT signals [29], we then assessed the relationship between ZIC5 and β-catenin expression. Unexpectedly, neither silencing nor overexpression of ZIC5 had an obvious effect on the expression levels of β-catenin, either in whole cells or in cell nuclei lysates (Fig. 3F, G). Consistent with these findings, immunofluorescence assays showed that ZIC5 overexpression barely altered the nuclear localization of β-catenin (Fig. 3H). These data indicated that ZIC5 induces Wnt/β-catenin signaling without affecting the nuclear translocation of β-catenin.

Subsequently, we addressed the mechanism by which ZIC5 regulates the transduction of Wnt signaling. Nuclear β-catenin binds to transcription factor 4 (TCF4) to form a β-catenin/TCF4 complex, which then activates the transcription of specific target genes [30]. To assess whether ZIC5 influences β-catenin/TCF4 complex formation, Co-IP assays were performed in C4-2B and 22RV1 cells. Results confirmed that ZIC5 co-immunoprecipitated with both β-catenin and TCF4 (Fig. 3I). Moreover, exogenous expression of β-catenin and TCF4 in 293T cells could be interact with ZIC5, respectively. (Supplementary Fig. 3D). To detect whether the β-catenin/TCF4 complex could be affected by ZIC5. Our results of Co-IP showed that ZIC5 strengthened β-catenin-TCF4 association in 293T (Fig. 3J, K). Collectively, these findings strongly suggest that ZIC5 promotes PCa metastasis by activating Wnt/β-catenin signaling via potentiating β-catenin/TCF4 complex formation.

### AR enhances ZIC5 expression through miR-27b-3p downregulation

**Fig. 4:**
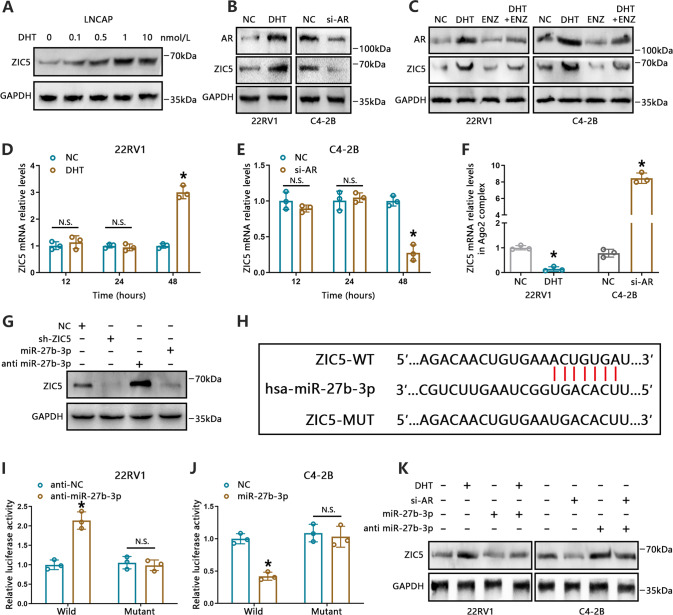
AR induces ZIC5 expression via miR-27b-3p downregulation. **A** LNCaP cells were cultured in charcoal-stripped serum medium for 3 days and then administered various doses of dihydrotestosterone (DHT) for 48 h. ZIC5 protein levels were detected using western blot. **B** Western blot analysis of AR and ZIC5 expression in 22RV1 cells treated with DHT (1 nmol/L) to activate AR and in C4-2B cells transfected with AR-specific siRNA (si-AR). **C** Western blot analysis of AR and ZIC5 expression in 22RV1 and C4-2B cells treated with or without DHT (1 nmol/L) and ENZ (10 μmol/L). **D**, **E** RT-qPCR analysis of relative ZIC5 mRNA levels in 22RV1 cells (**D**) treated with DHT (1 nmol/L) and in C4-2B cells (**E**) transfected with si-AR. N.S., *P* > 0.05 vs. NC, *P < 0.05, vs. NC. **F** 22RV1 cells treated with DHT (1 nmol/L) and C4-2B cells transfected with si-AR were immunoprecipitated with an Ago2 antibody, and relative ZIC5 mRNA levels in the Ago2 complex were detected by RT-qPCR. *P < 0.05, vs. NC. **G** Western blot analysis of ZIC5 expression following transfection of 22RV1 cells with sh-ZIC5, miR-27b-3p inhibitors, and miR-27b-3p mimics. **H** Potential miRNA-27b-3p binding sites on the ZIC5 3′-UTR. **I**, **J** Luciferase activities were determined in 22RV1 cells cotransfected with wild or mutant ZIC5 3′UTR vectors and miR-27b-3p inhibitors, and in C4-2B cells cotransfected with wild or mutant ZIC5 3′UTR vector and miR-27b-3p mimics. *P < 0.05, vs. NC, N.S., *P* > 0.05 vs. NC. **K** Western blot analysis of ZIC5 expression in 22RV1 cells treated with or without DHT and transfected with miR-27b-3p mimics, and in C4-2B cells cotransfected with or without si-AR and miR-27b-3p inhibitors.

To explore the mechanism responsible for ZIC5 upregulation in PCa, various pathways potentially involved were investigated. Given the key role of AR in PCa progression and its positive correlation with ZIC5 (Supplementary Fig. 4A). Further suggesting a possible link between AR and ZIC5 expression in PCa, we noticed that ZIC5 levels were higher in AR-positive than in AR-negative PCa cells (Fig. 1G). Next, androgen-sensitive LNCaP cells were cultured in charcoal-stripped serum medium for 3 days and then administered various doses of dihydrotestosterone (DHT) to stimulate AR signaling. Western blotting showed a strong upregulation of ZIC5 expression following stimulation with 1 nmol/L DHT (Fig. 4A). We then performed AR knockdown in C4-2B cells and induced DHT-mediated AR expression in 22RV1 cells to determine the influence of AR on ZIC5 expression. Consistent with the above findings, ZIC5 protein expression was reduced in C4-2B cells but was elevated instead in 22RV1 cells (Fig. 4B). Moreover, co-treatment with enzalutamide (Enz), a second-generation AR pathway antagonist, inhibited DHT-mediated ZIC5 expression in both C4-2B and 22RV1 cells (Fig. 4C).

Then, we focused on possible mechanisms underlying AR-dependent ZIC5 expression. Because ZIC5 mRNA levels were clearly altered by AR at 48 h, but not at 12 or 24 h compared to controls (Fig. 4D, E and Supplementary Fig. 4B). we speculated that AR modulates ZIC5 expression through a post-transcriptional mechanism. Considering the critical role of miRNAs in post-transcriptional regulation, an Ago2 antibody was used to pull down endogenous miRNA-ZIC5 complexes. Suggesting that AR-induced ZIC5 expression is indeed modulated by miRNA-ZIC5 interactions, assay results showed that ZIC5 mRNA levels in the Ago2 complex were reduced in DHT-treated 22RV1 cells but increased instead in AR-silenced C4-2B cells (Fig. 4F).

We next searched for potential ZIC5-binding miRNAs in multiple databases, including miRanda, PicTar, TargetScan, and PITA, accessed through the ENCORI platform. Search results consistently indicated that miR-27b-3p was a main predicted candidate. Based on this prediction, we interrogated TCGA data in the ENCORI platform and found that miR-27b-3p expression was downregulated in PCa, and its levels were inversely correlated with those of AR and ZIC5 (Supplementary Fig. 4D–F). Subsequently, we conducted RT-qPCR assays that showed that miR-27b-3p levels were elevated in AR-knockdown C4-2B cells and reduced instead in AR-stimulated 22RV1 cells (Supplementary Fig. 4C). Importantly, western blot analysis demonstrated that ZIC5 expression levels were notably reduced following transfection with miR-27b-3p mimics and increased, in turn, after miR-27b-3p inhibition (Fig. 4G).

Since miRNAs characteristically repress protein expression by binding to the 3′UTR of target mRNAs [31]. we next assayed a luciferase reporter vector containing putative miR-27b-3p binding sites in the 3′UTR of ZIC5. As shown in Fig. 4H–J, deletion of miR-27b-3p in 22RV1 cells led to upregulation of wild-type ZIC5-3′UTR luciferase activity, while the activity of a mutant ZIC5-3′UTR luciferase reporter was not altered. Conversely, transfection of miR-27b-3p mimics significantly reduced luciferase activity in C4-2B cells transfected with the wild-type, but not with the mutant, ZIC5-3′UTR reporter. Furthermore, western blot assays revealed that the introduction of miR-27b-3p mimics markedly diminished AR activation-induced upregulation of ZIC5 in 22RV1 cells, whereas miR-27b-3p inhibition reversed AR-reduced ZIC5 expression in C4-2B cells (Fig. 4K). The above data indicate that AR activation inhibits miR-27b-3p expression, resulting in enhanced translation of ZIC5 mRNA in PCa.

### AR association with SRC-3 modulates the transcription of miR-27b-3p

**Fig. 5:**
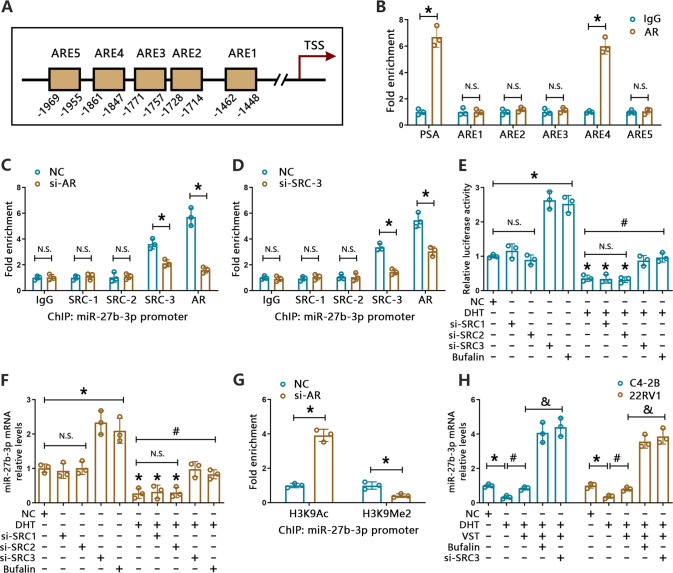
AR directly regulates the transcription of miR-27b-3p. **A** Putative androgen response elements (AREs) on the miR-27b-3p promoter region. **B** ChIP analysis of AR occupancy on the promoter region of miR-27b-3p in C4-2B cells. Purified IgG was used as control. *P < 0.05 vs. IgG. PSA were used as positive controls. **C** ChIP-qPCR analysis to assess specific binding of SRC-1, SRC-2, SRC-3, and AR to the miR-27b-3p promoter in C4-2B cells transfected with control or AR-targeted siRNA. Purified IgG was used as control. *P < 0.05 vs. NC, N.S. *P* > 0.05 vs. NC. **D** ChIP-qPCR analysis to assess specific binding of SRC-1, SRC-2, SRC-3, and AR to the miR-27b-3p promoter in 22RV1 cells transfected with control or SRC-3-targeted siRNA. Purified IgG was used as control. *P < 0.05 vs. NC, N.S. *P* > 0.05 vs. NC. **E** Luciferase-based detection of miR-27b-3p promoter activity in C4-2B cells exposed to the indicated treatments. *P < 0.05 vs. NC, ^#^*P* < 0.05 vs. DHT, N.S. *P* > 0.05 vs. NC or DHT. **F** RT-qPCR analysis of relative miR-27b-3p levels in C4-2B cells treated with or without DHT following administration of bufalin (50 nM, 24 h) or indicated SRC-targeted siRNA (48 h). *P < 0.05 vs. NC, ^#^*P* < 0.05 vs. DHT, N.S. *P* > 0.05 vs. NC or DHT. **G** ChIP-qPCR analysis to assess specific binding of H3K9Ac and H3K9Me2 to the miR-27b-3p promoter in C4-2B cells transfected with control or AR-targeted siRNA. Purified IgG was used as control. *P < 0.05 vs. NC. **H** RT-qPCR analysis of relative miR-27b-3p levels in C4-2B and 22RV1 cells treated with or without DHT following administration of VST (1 μM, 24 h) or bufalin (50 nM, 24 h) or SRC3-targeted siRNA (48 h). *P < 0.05 vs. NC, ^#^*P* < 0.05 vs. DHT, ^&^*P* < 0.05 vs. DHT + VST.

Since previous evidence implied that AR exerts transcriptional regulation of microRNAs in PCa [32, 33], we hypothesized that AR might bind to the promoter region of the miR-27b-3p gene to regulate its transcription. Bioinformatics analysis revealed five potential androgen-response-elements (AREs) on the promoter region of miR-27b-3p (Fig. 5A). Thus, those five AREs were selected for ChIP assay. We found obvious enrichment of AR in the ARE4 of the miR-27b-3p promoter (1847 to 1861 bp upstream of the TSS) but not on the other AREs (Fig. 5B). In addition, luciferase reporter assays in C4-2B and 22RV1 cells showed that siRNA-mediated AR inhibition drastically increased, whereas DHT-induced AR stimulation markedly inhibited, luciferase activity of the miR-27b-3p promoter. In contrast, neither inhibition nor stimulation of AR altered the activity of a mutant miR-27b-3p promoter in the above cell lines (Supplementary Fig. 5A). These results indicated that AR binds to the promoter of miR-27b-3p to repress its expression in PCa cells. The steroid receptor coactivator family (SRC-1, SRC-2, and SRC-3) has been well documented to interact with AR and regulate gene expression [34, 35]. However, whether this mechanism involves AR-mediated microRNA regulation remains uncertain. To verify the potential impact of SRCs on miR-27b-3p transcription in PCa cells, we first evaluated the SRCs expression in PCa via the GSE6919 and GSE3325 datasets. It was found that SRC-3 expression levels were obviously upregulated in metastatic PCa relative to localized carcinomas (Supplementary Fig. 5B). Similarly, analysis of TCGA-PCa dataset through GEPIA platform showed a positive correlation between AR and SRC-3 (NCOA3) (Supplementary Fig. 5C).

Next, ChIP assays using SRC-1, SRC-2, and SRC-3 antibodies were performed, and the results showed that only SRC-3 was notably enriched in the promoter of miR-27b-3p compared with IgG group (Fig. 5C). Furthermore, its occupancy could be strongly declined by AR knockdown (Fig. 5C). Likewise, SRC-3 depletion resulted in the erasure of AR binding on the miR-27b-3p promoter (Fig. 5D), indicating that binding of the coregulator stabilized the AR-complex on miR-27b-3p. In addition, neither AR nor SRC-3 showed obvious occupancy of the ZIC5 promoter, suggesting indirect modulation of ZIC5 by the AR (Supplementary Fig. 5D, E). To further ascertain the contribution of SRC-3 to AR-mediated miR-27b-3p transcriptional repression, SRCs were either inhibited, using bufalin (a pharmaceutical agent that selectively degrades SRC-1 and SRC-3) [36] or silenced, via specific siRNAs. Luciferase assay revealed that application of SRC-3 siRNA or bufalin, but not SRC-1 and SRC-2 depletion, was able to reduce AR activation-elicited repression of miR-27b-3p promoter activity in C4-2B and 22RV1 cells (Fig. 5E, Supplementary Fig. 5F). In parallel experiments, RT-qPCR confirmed that SRC-3 inhibition or depletion could increase the expression of miR-27b-3p. Moreover, the suppressive effect of AR activation on miR-27b-3p expression was relieved upon bufalin treatment or SRC-3 knockdown (Fig. 5F, Supplementary Fig. 5G). These findings demonstrated that SRC-3 associates with AR to prevent miR-27b-3p transcription in PCa cells.

Previous evidence revealed that AR or SRCs are able to recruit histone deacetylase families to exert gene repression functions [34, 37]. Moreover, our ChIP analysis with H3K9Ac (active histone mark) and H3K9Me2 (inactive histone mark) antibodies disclosed a strong occupancy of H3K9Ac at the miR-27b-3p promoter, while a significant decrease of H3K9Me2 at the miR-27b-3p promoter, after AR knockdown (Fig. 5G, Supplementary Fig. 5H). Thus, the impacts of pan-HDAC inhibitor vorinostat (VST) on miR-27b-3p expression were evaluated by RT- qPCR, and the results displayed that VST obviously rescued AR activation-mediated repression of miR-27b-3p, and this effect was further enhanced after combined treatment with bufalin (Fig. 5H). Our data revealed that AR/SRC-3 complex dependent transcriptional modulation may be achieved through the recruitment of HDACs to the miR-27b-3p promoter.

Subsequently, we assessed whether AR-mediated miR-27b-3p modulate ZIC5 levels to influence metastasis potential in PCa. We found that AR silencing repressed cell migration and invasion of C4-2B cells, and either application of miR-27b-3p inhibitors or ZIC5 overexpression reversed this effect (Supplementary Fig. 6A, B). Contrarily, AR stimulation increased the migration and invasion potential of 22RV1 cells, and this effect was abolished by miR-27b-3p mimics or ZIC5 inhibition (Supplementary Fig. 6C, D). Moreover, miR-27b-3p-elicited suppression of migration and invasion could be ameliorated by ZIC5 overexpression. Collectively, these findings showed that AR represses the transcription of miR-27b-3p to sustain ZIC5 expression, facilitating metastasis of PCa cells.

### ZIC5 elevates AR expression and potentiates resistance to enzalutamide in PCa cells

**Fig. 6:**
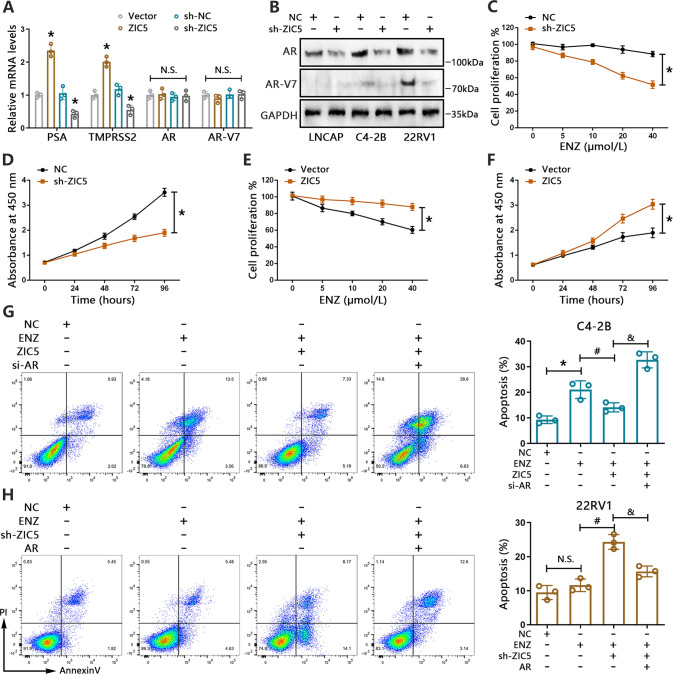
ZIC5 promotes enzalutamide resistance in PCa cells. **A** RT-qPCR analysis of AR target genes (PSA, TMPRSS2), AR, and AR-V7 in 22RV1 cells transfected with sh-ZIC5 or oe-ZIC5 for 48 h. *P < 0.05 vs. control vector or sh-NC, N.S. *P* > 0.05 vs. control vector. **B** Western blot analysis of AR and AR-V7 levels in LNCAP, C4-2B, and 22RV1 cells transfected with sh-ZIC5 or sh-NC for 72 h. **C**, **D** Results of CCK8 cell proliferation assays conducted on (**C**) 22RV1 cells transfected with sh-ZIC5 or NC shRNA and treated with various concentrations of enzalutamide (ENZ) for 72 h, and on (**D**) 22RV1 cells transfected with sh-ZIC5 or NC shRNA and treated with ENZ (20 μmol/L) for 24, 48, 72 or 96 h. *P < 0.05 vs. NC. **E**, **F** Results of CCK8 cell proliferation assays conducted on (**E**) C4-2B cells transfected with oe-ZIC5 or vector plasmids and treated with various concentrations of ENZ for 72 h, and on (**F**) C4-2B cells transfected with oe-ZIC5 or vector plasmids and treated with ENZ (20 μmol/L) for 24, 48, 72 or 96 h. *P < 0.05 vs. control vector. **G** Flow cytometry analysis of apoptosis in C4**-**2B cells treated with ENZ (20 μmol/L) for 72 h and transfected, as indicated, with oe-ZIC5, si-AR. The bar graph shows quantification data from three independent experiments. *P < 0.05 vs. NC, ^#^*P* < 0.05 vs. ENZ, ^&^*P* < 0.05 vs. ENZ + ZIC5. **H** Flow cytometry analysis of apoptosis in 22RV1 cells treated with ENZ (20 μmol/L) for 72 h and transfected, as indicated, with sh-ZIC5, AR expression plasmids. The bar graph shows quantification data from three independent experiments. N.S., *P* > 0.05 vs. NC, ^#^*P* < 0.05 vs. ENZ, ^&^*P* < 0.05 vs. ENZ + sh-ZIC5.

AR modulates the expression of many androgen-response gene products [38], several of which may in turn influence AR expression and activation of AR signaling [39, 40]. In our study, we found that AR could augment ZIC5 levels in PCa cells. However, whether AR could be altered by ZIC5 is still uncertain. To test our hypothesis, three AR-positive cell lines (LNCAP, C4-2B and 22RV1) were used in our analysis. Genetic overexpression or inhibition of ZIC5 in LNCAP, C4-2B and 22RV1 cells caused a notably increase or decrease in the mRNA levels of AR target genes including PSA and TMPRSS2, respectively (Fig. 6A and Supplementary Fig. 7A, B). Nevertheless, ZIC5 had no significant effect on the mRNA levels of AR and AR-V7 (Fig. 6A and Supplementary Fig. 7A, B). Next, we performed western blot assays to determine whether ZIC5 levels influence the expression of AR and AR-splice variant 7 (AR-V7) protein in PCa cells. Suggesting a stimulatory effect of ZIC5 on AR expression and signaling in PCa cells, our results confirmed a decline in AR protein levels upon ZIC5 depletion, as well as downregulation of AR-V7 (Fig. 6B).

Compelling evidence indicates that sustained AR activity is one of the essential causes of PCa resistance to enzalutamide (Enz) [41]. We thus posited that ZIC5-induced AR expression might contribute to Enz resistance in PCa. Cell proliferation assays on C4-2B and 22RV1 cells treated with various doses of Enz revealed that ZIC5 silencing compromised Enz resistance by reducing viability in 22RV1 cells, while ZIC5 overexpression alleviated Enz-mediated growth suppression in C4-2B cells (Fig. 6C–F). Importantly, we found that Enz application alone or in combination with ZIC5 depletion dramatically impaired the colony formation capacity of C4-2B cells, and this effect was reduced by AR overexpression (Supplementary Fig. 7C, D). Similarly, EdU assays showed that forced AR expression notably weakened the inhibitory effect of combined Enz treatment and ZIC5 knockdown on the proliferation of 22RV1 cells (Supplementary Fig. 8A, B). In turn, ZIC5-overexpressing C4-2B cells showed less apoptosis in response to Enz, an effect reversed by AR inhibition (Fig. 6G). Conversely, ZIC5 silencing increased apoptosis in 22RV1 cells treated with Enz, and this effect was diminished upon AR overexpression (Fig. 6H). These results cumulatively suggest that ZIC5 induces Enz resistance in PCa cells by enhancing AR expression.

### ZIC5 inhibition increases the sensitivity of PCa to enzalutamide in mice

**Fig. 7:**
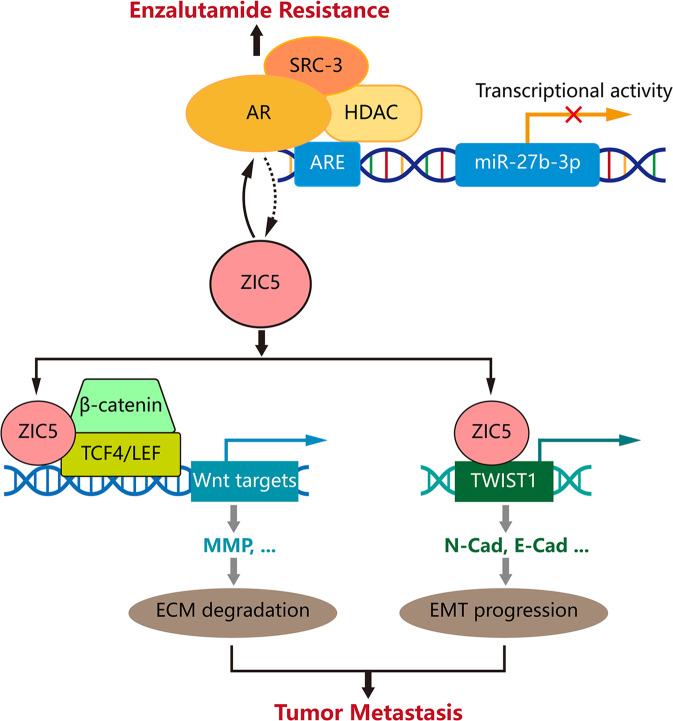
Mechanistic model of AR-ZIC5 axis-induced metastasis and enzalutamide resistance in PCa. Following AR/SRC-3/miR-27b-3p axis-induced expression, ZIC5 contributes to PCa metastasis by acting as a transcription factor, to promote TWIST1 transcription and EMT progression, or as a cofactor, to stimulate Wnt/β-catenin signaling and extracellular matrix (ECM) degradation. In parallel, ZIC5 sustains AR expression and signaling, thus favoring the development of enzalutamide resistance.

To recapitulate the findings of the cell experiments described above, the impact of ZIC5 expression was examined using PCa xenografts. 22RV1 cells (Enz-insensitive PCa cell line) transfected with ZIC5-targeted shRNA or control shRNA were injected subcutaneously into nude mice, divided into four groups to receive Enz or saline (control). As shown in Supplementary Fig. 9A, there was no significant difference in 22RV1 tumor size between control and Enz-treated mice. However, ZIC5 depletion led to a reduction in tumor growth, and this effect was enhanced by the combination of Enz treatment and ZIC5 knockdown. Parallelly, the tumor weight and tumor volume revealed a similar trend (Supplementary Fig. 9B, C). Consistent with these findings, IHC staining revealed lower Ki-67 expression in tumors from ZIC5-inhibited mice, and the combination of Enz treatment and ZIC5 knockdown strengthened this antiproliferative effect (Supplementary Fig. 9D, E). These data indicate that ZIC5 inhibition increases the efficacy of Enz against PCa growth in vivo. Finally, a schematic model depicting the proposed mechanism responsible for AR-ZIC5 axis-mediated metastasis and resistance to Enz in PCa is shown in Fig. 7.

## Discussion

Prostate cancer remains one of the most common and heterogeneous malignancies in men. Despite initial sensitivity to androgen deprivation therapy (ADT), almost all patients inevitably progress to ADT resistance and develop castration-resistant PCa (CRPC), sometimes in combination with tumor metastases [42, 43]. Hence, developing effective treatments to prevent the progression of PCa to advanced stages remains imperative. Accumulating evidence shows that ZIC5 is dysregulated in various cancers and correlates with outcome and prognosis of patients [19, 44, 45]. ZIC5 was proposed to support cancer cell growth, invasion, and migration, and its expression was associated with drug resistance and tumor metastasis [22–25]. In the studies of PCa, Satow et al. and Hoogland et al. reported that ZIC5 was upregulated in high-grade PCa tumor tissues and was positively associated with gleason score [21, 46]. Besides, Satow and colleagues revealed that knockdown of ZIC5 suppressed AR-negative PCa cell proliferation through PDGFD/FAK/STAT3 signaling [21]. However, neither Satow et al. [21] nor Wang et al. [47] succeeded to elucidate the expression levels of ZIC5 in PCa cell lines (e.g., AR-positive PCa cells) and the role played by ZIC5 in PCa tumor aggressiveness. Indeed, we observed that ZIC5 levels were significantly increased in AR-positive cell lines and human PCa samples, and this expression pattern correlated negatively with overall survival. Further suggesting the carcinogenic potential of dysregulated ZIC5 expression in PCa, we detected higher levels of ZIC5 expression in metastatic, relative to localized, PCa specimens. Moreover, depleting ZIC5 markedly attenuated the migration and invasion and colony formation capacities of C4-2B and 22RV1 cells. Interestingly, ZIC5 had a limited effect on the invasion and migration ability of AR-negative PC3 cells. Only forced ZIC5 expression in PC3 cells could slightly augment cell metastasis, probably because PC3 cells harbored little AR and ZIC5. However, this also suggests, to some extent, that AR-ZIC5 coactivation promotes PCa cell metastasis. Still, our findings indicated that ZIC5 upregulation was associated with metastatic status in PCa patients and that ZIC5 might also be a prognostic marker for patients suffering from metastatic PCa, as well as a relevant therapeutic target.

In exploring the possible mechanisms of ZIC5-mediated metastasis in PCa cells, we found that EMT progression and Wnt/β-catenin signaling activation were closely associated with ZIC5. EMT programs have been described as essential events in PCa progression, metastasis, and enhanced stemness of cancer stem cells, as well as in induction of castration-resistance in PCa patients [48]. Moreover, ZIC5 was reported to drive melanoma EMT progression and metastasis through transcriptional downregulation of E-cadherin expression [25]. Consistent with those findings, our study revealed that ZIC5 overexpression notably potentiated EMT progression in PCa cells, as evidenced by upregulation of mesenchymal markers and downregulation of epithelium-related proteins. Activation of the EMT program in primary cancer cells is orchestrated by various EMT-inducing transcription factors (e.g. TWIST families) [27]. TWIST1 enhances the ability of primary tumor cells to undergo EMT and thus promotes invasive and metastatic cancer phenotypes [49]. In PCa, upregulation of TWIST1 through Sox5 increased the migration capacity and mesenchymal phenotype of cancer cells, initiated the EMT program, and promoted tumor lymph node metastasis [50]. In this work, ZIC5 acted as a transcription factor that bound to the TWIST1 promoter region, while ZIC5 inhibition clearly attenuated TWIST1 expression and reduced its transcriptional activity, resulting in EMT repression. Thus, our data indicated that ZIC5 promotes metastasis of PCa cells through positive regulation of TWIST1-induced EMT progression. However, whether transcriptional cofactors intervene in the regulation of TWIST1 by ZIC5 needs further assessment.

Abnormal Wnt/β-catenin signaling has significant effects on cancer progression and metastasis [28]. Upon nuclear translocation, β-catenin binds to TCF/LEF family members to activate downstream genes that drive metastatic progression [51]. Recent studies showed that ZIC5 modulates Wnt/β-catenin signaling to influence the growth and metastasis of hepatocellular carcinoma cells and glucose metabolism in colorectal cancer [20, 23]. In line with these studies, we revealed that ZIC5 overexpression led to significant upregulation of Wnt/β-catenin signaling targets, including c-Myc, MMP2, and MMP7. We further noticed that the β-catenin agonist LiCl ameliorated the suppression of Wnt/β-catenin signaling elicited by ZIC5 knockdown. Moreover, the inhibitory effect of ZIC5 depletion on PCa cell migration and invasion was blunted by LiCl, further proving that Wnt/β-catenin signaling is intimately involved in the metastasis-promoting effect of ZIC5. Nevertheless, we observed that ZIC5 promoted the transduction of Wnt signals without affecting the nuclear localization of β-catenin. Instead, Co-IP assays revealed that ZIC5 served as a cofactor to interact with the β-catenin/TCF4 complex and strengthened complex formation, a necessary step leading to downstream gene transcription [30, 52–54]. Supporting this evidence, a previous study showed that ZIC5 interacts with the β-catenin/TCF4 complex to repress GLUT1 expression and regulate glucose metabolism in HCT 116 colon cancer cells [20]. Therefore, the anti-metastatic effect of ZIC5 inhibition in PCa cells can also be attributed to repressed Wnt/β-catenin signaling.

Androgen/AR signaling significantly impacts the modulation of genes involved in PCa proliferation, progression, metastasis, and therapy resistance [13, 55, 56]. In canonical AR signaling, after binding to its ligand androgen, the AR residing in the cytoplasm dissociates from heat shock proteins and is subsequently translocated to the nucleus. Following dimerization and activation, AR binds to AREs in the target gene promoter to initiate gene transcription [57]. A recent study showed that AR activation promotes the expression of the cell cycle regulatory protein FAM64A, leading to increased migration and invasion of PCa cells [12]. Another study indicated that AR-mediated upregulation of TMPRSS2 enhances PCa growth and metastasis through activation of matriptase and promotion of extracellular matrix (ECM) degradation [9]. Since the expression of AR was positively correlated with that of ZIC5 in TCGA-PCa patient samples and ZIC5 levels were higher in AR-positive than in AR-negative PCa cells, we carried out gain- and loss-of-function experiments that confirmed that AR stimulates ZIC5 expression, thereby strengthening PCa cell migration and invasion abilities. Because we observed delayed stimulation of ZIC5 expression following AR signaling induction (i.e. by 48 h after AR alteration), we hypothesized that an indirect, miRNA-mediated mechanism, might be involved. Following bioinformatics prediction analysis, ChIP and gene reporter assays uncovered that AR was enriched on the promoter of miR-27b-3p and transcriptionally repressed miR-27b-3p to maintain the upregulation of ZIC5. These results thus identify a novel signaling axis, AR/miR-27b-3p/ZIC5, with a potentially major impact on PCa metastasis.

Mounting evidence indicates that the transcriptional activity of AR requires structural and functional interactions with several coregulators [34, 57]. SRC-3, an essential coregulator of AR, has been reported to be significantly elevated and amplified in PCa patients. Abnormally high levels of SRC-3, in association with increasing PSA levels and enhanced tumor cell proliferation, were identified in PCa, which led to propose SRC-3 as an independent marker of tumor recurrence [58]. In this study, we discovered that SRC-3 was recruited to the miR-27b-3p promoter, and AR silencing abated this occupancy. Further, SRC-3 blockade, via bufalin-mediated inhibition or siRNA-mediated silencing, was able to abolish AR-driven repression of miR-27b-3p. Of note, A abolition effect can be further strengthened when concomitantly administered with HDAC inhibitors, which indicated that AR/SRC-3 complex dependent transcriptional repression of miR-27b-3p may be mediated through recruitment of HDACs. Recent studies have demonstrated that HDACs mediate the genetic repression function of SRCs [37, 59]. Histone deacetylases (HDACs), which remove acetyl groups from histone tail lysine residues, trigger a more condensed chromatin structure, resulting in gene repression [60]. Indeed, various HDACs have been shown to influence cancer progression via the regulation of miRNA expression. For instance, through the recruitment of HDAC, PELP1 was shown to silence the expression of miR-200 family members and to promote breast cancer metastasis [61]. Consistent with these findings, and based on our data, we uncovered that transcriptional inhibition of miR-27b-3p induced by the AR/SRC-3 complex might be mediated by recruiting HDACs. Taken together, these studies attest that the AR/SRC-3 complex interacts with HDACs to transcriptionally suppress miR-27b-3p expression, which sustains high ZIC5 expression to promote PCa cell metastasis.

The occurrence of bidirectional cross talk and mutual regulation between AR and target genes or proteins, as well as with other signaling pathways, is widely recognized [62–64]. For instance, AR can bind to the ARE region of the MAOA gene to induce it expression, which in turn potentiates the transcriptional activity of AR through induction of AR/YAP1 interaction [39]. Meanwhile, another study in PCa cells indicated that KIF4A expression, regulated by AR, stabilized AR and AR-V7 to trigger Enz resistance [65]. In line with these findings, our observations support the existence of a positive feed-forward loop between AR signaling and ZIC5 involving AR-mediated downregulation of miR-27b-3p, subsequent enhancement of ZIC5 mRNA translation, and ZIC5-induced AR signaling. Accordingly, we showed that ZIC5 knockdown led to downregulation of AR targets, and AR, AR-V7 protein expression in vitro, and amplified the antitumor efficacy of Enz in PCa xenografts in vivo. These findings demonstrated that ZIC5 potentiates the expression of AR and AR targets in PCa cells, contributing to Enz resistance. In addition, it was observed a reduction in AR and AR-V7 proteins but not AR transcripts after ZIC5 knockdown, which indicated that ZIC5 modulates AR protein stability through non-transcriptional mechanisms. Indeed, a recent report proves that ZIC5 interacts with Gli3 to sustain Gli3 protein stabilization through the decrease of Gli3 ubiquitination [66]. However, whether this mechanism contributes to the regulation of AR by ZIC5 requires further exploration.

In summary, our work demonstrated that aberrantly high levels of ZIC5 correlated with poor survival and metastatic status in PCa patients and that its expression was regulated by AR. On the one hand, AR cooperated with SRC-3 to increase ZIC5-mediated PCa cell metastasis through repression of miR-27b-3p transcription. On the other hand, ZIC5 contributed to enzalutamide treatment resistance by elevating AR signaling. Therefore, we uncovered a novel positive feed-forward loop linking AR and ZIC5 in PCa tumor malignant progression. Further, ZIC5 inhibition prevented AR activation-induced PCa cell metastasis and enhanced the sensitivity of PCa to enzalutamide. Thus, cotargeting ZIC5 and AR might represent a more effective therapy for advanced PCa patients.

## Materials and methods

### Cell culture and reagents

The PCa cell lines LNCAP, C4-2B, and 22RV1 were obtained from the American Type Culture Collection (Manassas, USA). The PCa cell lines DU145 and PC3, as well as the normal human prostate epithelial cell line RWPE1, were purchased from Cell Bank of the Chinese Academy of Sciences (Shanghai, China). The PC3 cell line was cultured in F12K media (GIBCO), and the other four PCa cell lines were maintained in RPMI-1640 medium. All culture media contained 1% penicillin-streptomycin and 10% fetal bovine serum (FBS; GIBCO). The RWPE1 cell line was cultured in keratinocyte serum-free medium (K-SFM) supplemented with bovine pituitary extract and human recombinant epidermal growth factor. All cells were incubated at 37 °C in a humidified atmosphere with 5% CO_2_. For dihydrotestosterone (DHT, Sigma-Aldrich, USA) treatments, phenol red-free media containing 10% charcoal-stripped FBS (GIBCO) was used instead. Vorinostat (VST, S1047) and enzalutamide (Enz, S1250) were purchased from Selleck Chem (USA). Bufalin (203900) were purchased from Sigma-Aldrich (USA).

### Clinical samples

This study was approved by the Institutional Ethics Committee of Huashan Hospital, Fudan University, and was conducted in accordance with the World Medical Association Declaration of Helsinki. Ten specimens of benign prostatic hyperplasia (BPH) and 10 pairs of PCa samples and adjacent non-cancerous tissue were collected from patients who had undergone surgical treatment at the Dept. of Urology, Huashan Hospital, Fudan University, between 2020 and 2021. In addition, five metastatic prostate tumor samples in distant sites from individual patients were obtained from the Dept. of Oncology, Sixth People’s Hospital affiliated to Shanghai Jiao Tong University. Specimens were immediately preserved in liquid nitrogen and stored at −80 °C until analysis. Two independent senior pathologists evaluated the histological characteristics of the samples and confirmed pathological diagnosis. All patients signed and provided informed consents.

### Bioinformatics analysis

The UCSC Xena platform (http://xena.ucsc.edu/) was accessed to explore the prognostic role of ZIC5 in PCa patients in TCGA database. The association between ZIC5 and either EMT markers and Wnt/β-catenin targets was investigated using TCGA-PCa patient data retrieved in the ENCORI (http://starbase.sysu.edu.cn) [67, 68] or the GEPIA (http://gepia.cancer-pku.cn) platforms. The GSE6919 and GSE3325 datasets were obtained from the Gene Expression Omnibus (GEO) (http://www.ncbi.nlm.nih.gov/geo).

### Reverse transcription-quantitative PCR

Total RNA was isolated with TRIzol reagent (Invitrogen, USA) according to the manufacturer’s protocol. The PrimeScript RT reagent kit (Takara, Japan) was used to synthesize complementary DNA. Quantitative reverse transcription PCR (RT-qPCR) was performed with SYBR Premix Ex Taq kit (Takara, Japan), utilizing an StepOnePlus Real-Time PCR System (Applied Biosystems, USA). GAPDH or U6 were used as internal controls, and relative gene expression was calculated by the 2-ΔΔCt method. Primer sequences are listed in Supplementary Table 1 and were all designed by Sangon Biotech (Shanghai, China).

### Immunohistochemical staining

Immunohistochemical staining (IHC) was carried out as we previously described [69]. Briefly, paraformaldehyde-fixed paraffin tissues were sectioned into 4-μm thick slides and deparaffinized and rehydrated according to standard protocols. Slides were incubated with primary antibodies overnight, washed, and incubated with horseradish peroxidase-conjugated secondary antibodies (Thermo, USA). Following reaction with 3,3-diaminobenzidine (Servicebio, China), sections were visualized and photographed with an Olympus BX40 microscopy (Tokyo, Japan). Primary antibodies included anti-ZIC5 (OAEB00858, Aviva Systems Biology, USA; bs-12147R, Bioss, China), and anti-KI67 (ab15580, Abcam). For quantitation, IHC scores were obtained according to the percentage of positive cells (0, <10%; 1, 11–25%; 2, 26–50%; 3, 51–75%; and 4, 76–100%) and the intensity of staining (0 = negative staining; 1 = weak staining; 2 = moderate staining; and 3 = strong staining). The final staining score was the product of these two scores.

### Cell proliferation assays

PCa cells (5000 cells/well) were seeded into 96-well plates for 24 h. After the indicated treatments, CCK8 reagent (Dojindo, Japan) was added into each well and incubated for 2 h at room temperature in the dark. A spectrophotometer was then used to determine absorbance at 450 nm.

### Colony formation assay

PCa cells were trypsinized, counted, seeded into six-well plates (1000 cells/well), and maintained in media supplemented with 10% FBS for 10 days. After fixation with 4% paraformaldehyde, cell colonies were visualized by staining with crystal violet solution (Beyotime, China) and counted under light microscopy. Clones (>50 cells/colony) were scored and divided by the total number of cells seeded to obtain the clone formation rate.

### Wound healing and cell invasion assays

PCa cells were cultured to confluence in six-well dishes in media containing 10% FBS, and pipette tips (200 μL) were employed to generate linear wounds. After the removal of detached cells, the supernatant was replaced with a medium containing 1% FBS. At indicated times, scratch gaps were photographed and measured. Corning Transwell chambers pre-coated with Matrigel (BD Bioscience, USA) were used for cell invasion assays. The upper chambers were seeded with 1 × 10^5^ cells resuspended in 200 µl of serum-free medium, while the lower chambers were filled with complete media (500 µl) containing 10% FBS. After 24 h of incubation, cells remaining in the upper chamber were removed, and cells invading the lower surface of the chamber were fixed with paraformaldehyde and stained with crystal violet solution (Beyotime, China). The number of invading cells was then determined by microscopy.

### Plasmid construction and transfection

Full-length ZIC5 cDNA was subcloned into flag-tagged pcDNA3.1 expression vectors as previously described [25]. For overexpression experiments, PCa cells were seeded into 6-well plates (2 × 10^5^ cells/well) and plasmid transfection was performed using Lipofectamine 2000 reagent in accordance with standard protocols. For ZIC5 knockdown assays, PCa cells were transfected with recombinant lentiviruses containing ZIC5-targeted shRNA (GenePharma, Shanghai, China) at MOI of 50 in serum-free medium. After 6 h, the media were replaced and cells were further incubated for another 48 h. Stable ZIC5 low-expression cells were selected with puromycin. AR-targeting and non-specific negative control siRNAs, constructed by GenePharma (Shanghai, China), miRNA-27b-3p mimics and inhibitors, as well as negative control miRNAs, obtained from Ribobio (Guangzhou, China) were transfected into PCa cells plated in six-well dishes using Lipofectamine 2000 as per the manufacturer’s manual.

### Flow cytometry analysis

Cellular apoptosis was evaluated in PCa cells using an Annexin V/PI apoptosis kit following the manufacturer’s instructions (Multisciences, China). Following treatments, the cells were collected and stained for 15 min with propidium iodide (5 µl) and annexin‐fluorescein isothiocyanate (10 μl) in the dark. A FACSCalibur flow cytometry instrument (BD, USA) was used to analyze apoptosis.

### 5-Ethynyl-2′-deoxyuridine (EdU) assay

Cell-Light Edu imaging detection kit purchased form RiboBio (Guangzhou, China), was carried out to measure cell proliferation as specified in the manufacturer protocol. In short, EdU reagents (10 µM) were added into the cells (per well) and incubated for 4 h. After paraformaldehyde fixation, cells were co-stained with Apollo fluorescent dyes and Hoechst 33342 (5 µg/ml). The images were captured with an Olympus fluorescence microscope (Tokyo, Japan).

### Western blotting

Total proteins were extracted with RIPA lysis buffer (Beyotime, China) and protein concentrations quantified by a BCA assay. Equal amounts of protein were separated by 8–12% SDS-PAGE (Servicebio, China) and transferred onto PVDF membranes (Servicebio, China). After blocking with 5% nonfat dry milk, the membranes were incubated with the following primary antibodies: anti-N-cadherin (ab18203, Abcam, UK), anti-E-cadherin (3195, Cell Signaling Technology, CST, USA), anti-Snail1 (3879, CST), anti-TWIST1 (25465-1-AP, Proteintech, USA), anti-ZIC5 (ARP33669-P050, Aviva Systems Biology, USA; bs-12147R, Bioss, China), anti-β-actin (ab8227, Abcam), anti-β-catenin (51067-2-AP, Proteintech), anti-TCF4 (22337-1-AP, Proteintech), anti-Histone H3 (ab1791, Abcam), anti-Tubulin (2148, CST), anti-AR-V7 (19672, CST), anti-AR (ab108341, Abcam), and anti-GAPDH (ab9485, Abcam). After incubation with suitable HRP-conjugated antibodies, the blots were visualized using an ECL kit (Multisciences, China).

### Luciferase reporter assay

TWIST1 and miR-27b-3p promoter-driven luciferase reporter and mutant vectors, as well as TCF/LEF and ZIC5 3′UTR luciferase reporter vectors were constructed by Sangon Biotech (Shanghai, China). PCa cells were transfected with ZIC5 shRNA or ZIC5 overexpression plasmid together with the TWIST1 promoter luciferase reporter or LEF luciferase vectors using Lipofectamine 2000 reagent according to the standard protocol. To assess the interaction between AR and the miR-27b-3p promoter, cells transfected with AR siRNA or pre-treated with DHT were transfected with miR-27b-3p luciferase reporter plasmids and Renilla luciferase vector for 48 h. Firefly luciferase activities were normalized against Renilla luciferase activities. All experiments were repeated three times and luciferase activity was detected using a Dual Luciferase Reporter Assay System (Promega, USA).

### Chromatin immunoprecipitation

ChIP assay was performed according to the established method. Briefly, following experimental treatments the cells were crosslinked with 1% formaldehyde and quenched by glycine (125 mM). After lysis in ChIP lysis buffer (Beyotime, China), sonication, and centrifugation at 4 °C for 10 min (10,000 rpm), the supernatant was obtained and then diluted with dilution buffer. Immunoprecipitation was then performed through application of anti-Flag (#14793, CST), anti-AR (ab108341, Abcam), or anti-SRC-1 (#2191, CST), anti-SRC-2 (#96687, CST), anti-SRC-3 (#2126, CST) or anti-H3K9Ac (PA5-117092, Thermo), anti-H3K9Me2 (PA5-120810, Thermo) antibodies, incubated overnight at 4 °C. Protein magnetic beads were used to collect immune complexes. Following additional washing, elution with elution buffer, and heating at 65 °C for 4 h for reverse cross-linking, the associated DNA fragments were purified and analyzed by RT-qPCR or agarose gel electrophoresis.

### Immunoprecipitation assay

IP experiments were performed following standard procedures. Briefly, PCa cells were transfected with the indicated plasmids, and nuclear proteins were isolated with a Nucleoprotein Extraction Kit (Sangon Biotech, Shanghai, China) following the manufacturer’s protocol. Protein samples were then incubated with primary antibodies at 4 °C for 4 h. Subsequently, pre-cleared protein A/G beads (Invitrogen, 88803) were added and the samples were further incubated at 4 °C overnight. Bead-protein complexes were precipitated by centrifugation and washed five times to remove nonspecific bound proteins. IP products were then boiled for 5 min in loading buffer and then identified through western blot analysis as described above.

### Immunofluorescence staining

PCa cells were seeded on glass coverslips in six-well plates. After adherence, cells were fixed in 4% paraformaldehyde for 15 min, permeated by Triton X-100 for 10 min at room temperature, and blocked with bovine serum albumin for 30 min. Next, the slides were incubated with anti-β-catenin antibody (51067-2-AP, Proteintech) overnight at 4 °C and subsequently incubated with a fluorescently labeled secondary antibody (Antgene, Wuhan, China) for 1 h at 37 °C. Nuclei were counterstained with DAPI and images captured by Olympus fluorescence microscopy (Tokyo, Japan).

### Animal assay

All animal studies were approved by the Animal Care and Use Committee of Fudan University and conformed to routine animal-care guidelines. Male BALB/c nude mice (aged 4–5 weeks, Vital River Laboratory Animal, China) were housed under specific pathogen-free conditions. To determine the effect of ZIC5 expression on enzalutamide efficacy in vivo, 1 × 10^6^ 22RV1 cells stably expressing ZIC5 shRNA (sh-ZIC5) or control shRNA (sh-NC) were resuspended in 100 µl PBS and implanted subcutaneously into the dorsal flanks of the mice. Then, animals were surgically castrated and divided into four experimental groups and treated with enzalutamide (Enz, 10 mg/kg orally) or vehicle control (physiological saline, PS, orally) as follows: (1) control (sh-NC + PS, orally); (2) Enz treatment (sh-NC + Enz, 10 mg/kg, orally); (3) ZIC5 inhibition (sh-ZIC5 + PS, orally); and (4) combined treatment (sh-ZIC5 + Enz, 10 mg/kg, orally). Tumor volume was monitored with Vernier calipers and calculated according to the formula: width^2 ^× length (mm^3^) × 0.5. Thirty days later, the mice were sacrificed.

### Statistical analysis

Statistical analyses were performed with GraphPad Prism 6 and SPSS 20.0 software. Data are presented as mean ± SD from three independent experiments. Student’s *t*-test was employed to assess differences between the two groups. One-way ANOVA and the Bonferroni post hoc test were used for multiple comparisons. *P* < 0.05 was considered significant.

## Supplementary information

Supplementary figure 1

Supplementary figure 2

Supplementary figure 3

Supplementary figure 4

Supplementary figure 5

Supplementary figure 6

Supplementary figure 7

Supplementary figure 8

Supplementary figure 9

Supplementary Figure legends

Supplementary Table 1

Western blot original data

## Data Availability

The original data generated were included in the present manuscript.
