# Supplementary figures and images for: AR-regulated ZIC5 contributes to the aggressiveness of prostate cancer
Source: Cell Death Discov. 2022 Sep 20;8:393. doi: 10.1038/s41420-022-01181-4 (PMC9489711; doi:10.1038/s41420-022-01181-4)

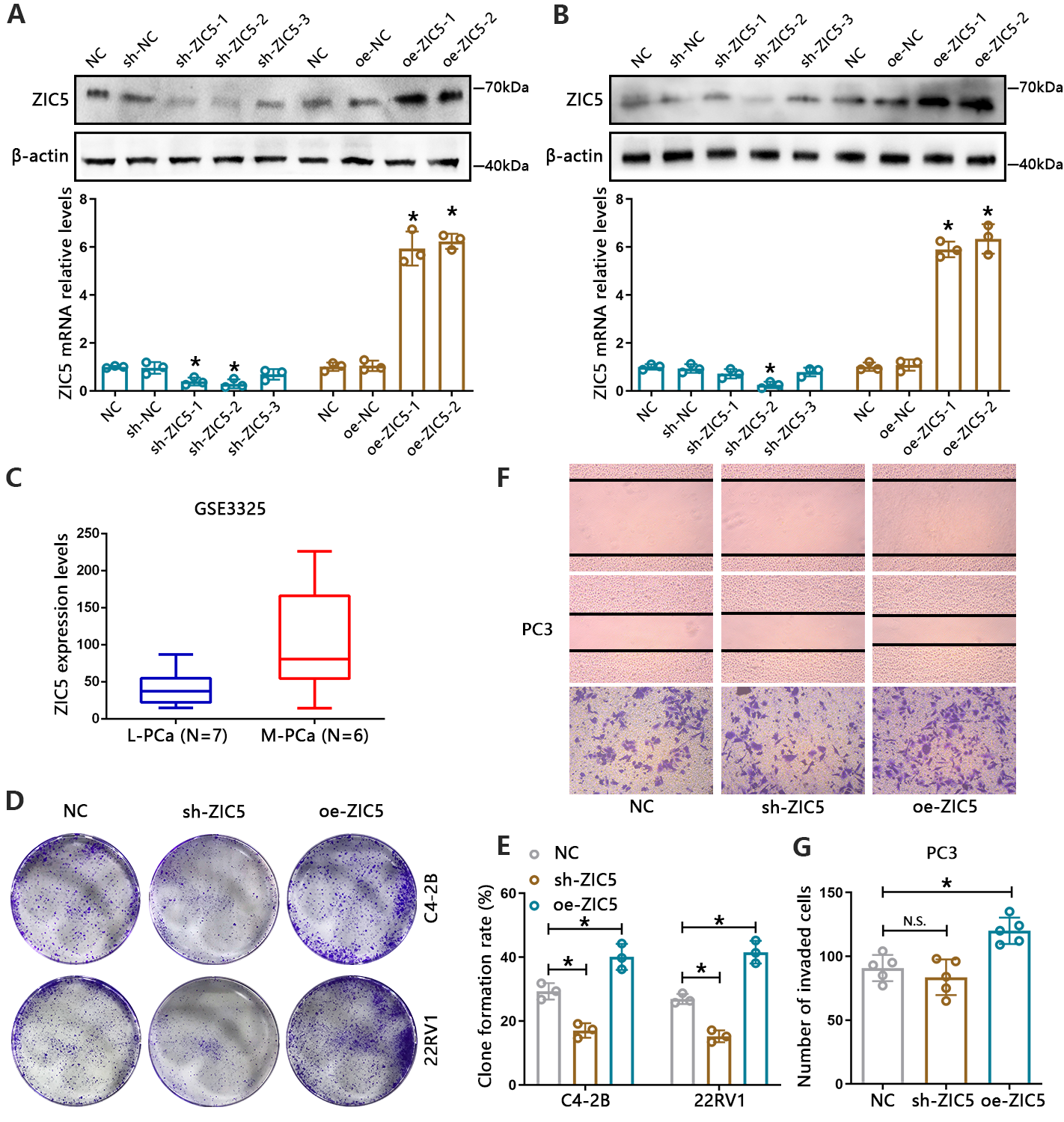

Supplement: Supplementary file 1 — Supplementary figure 1 [file 41420_2022_1181_MOESM1_ESM.tif]

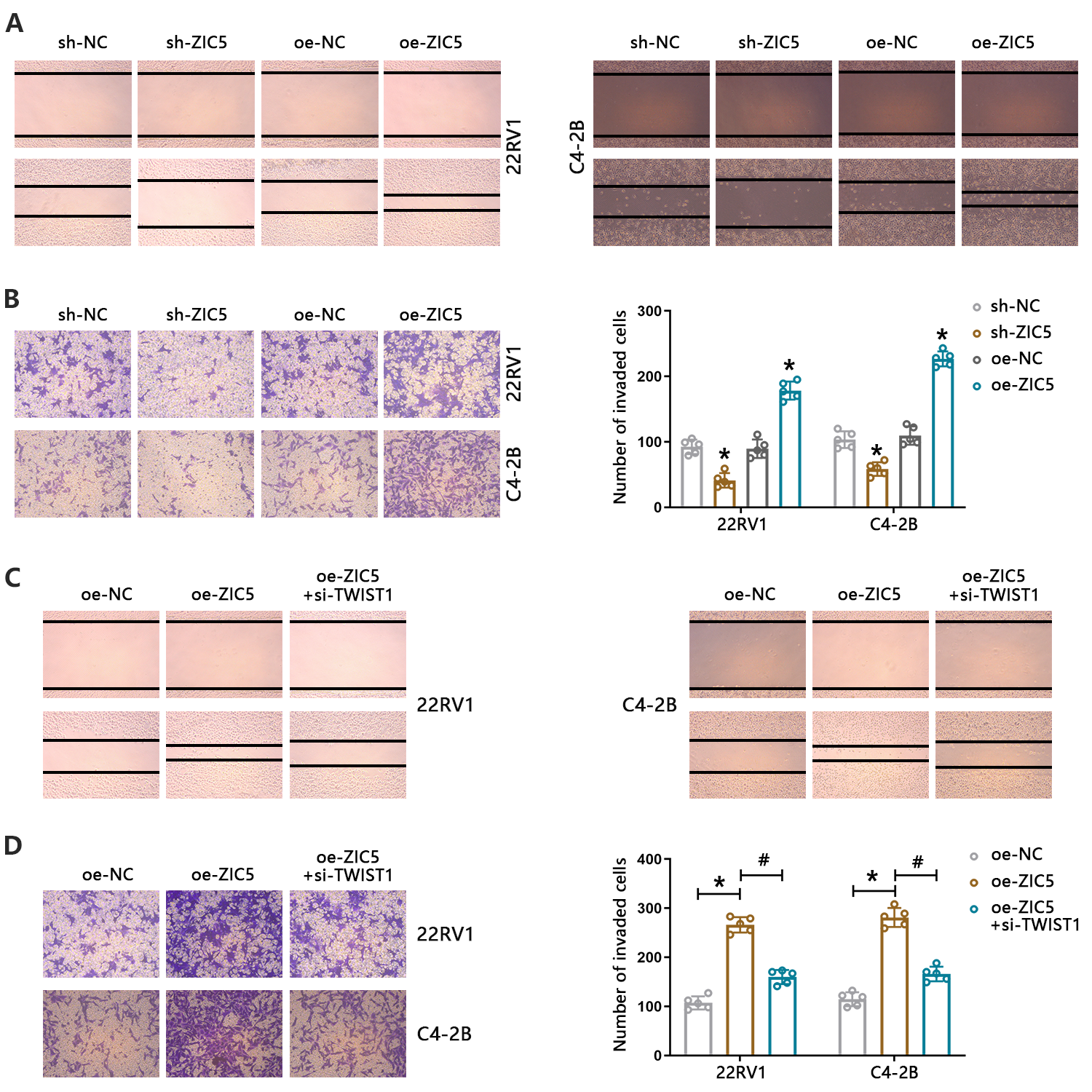

Supplement: Supplementary file 2 — Supplementary figure 2 [file 41420_2022_1181_MOESM2_ESM.tif]

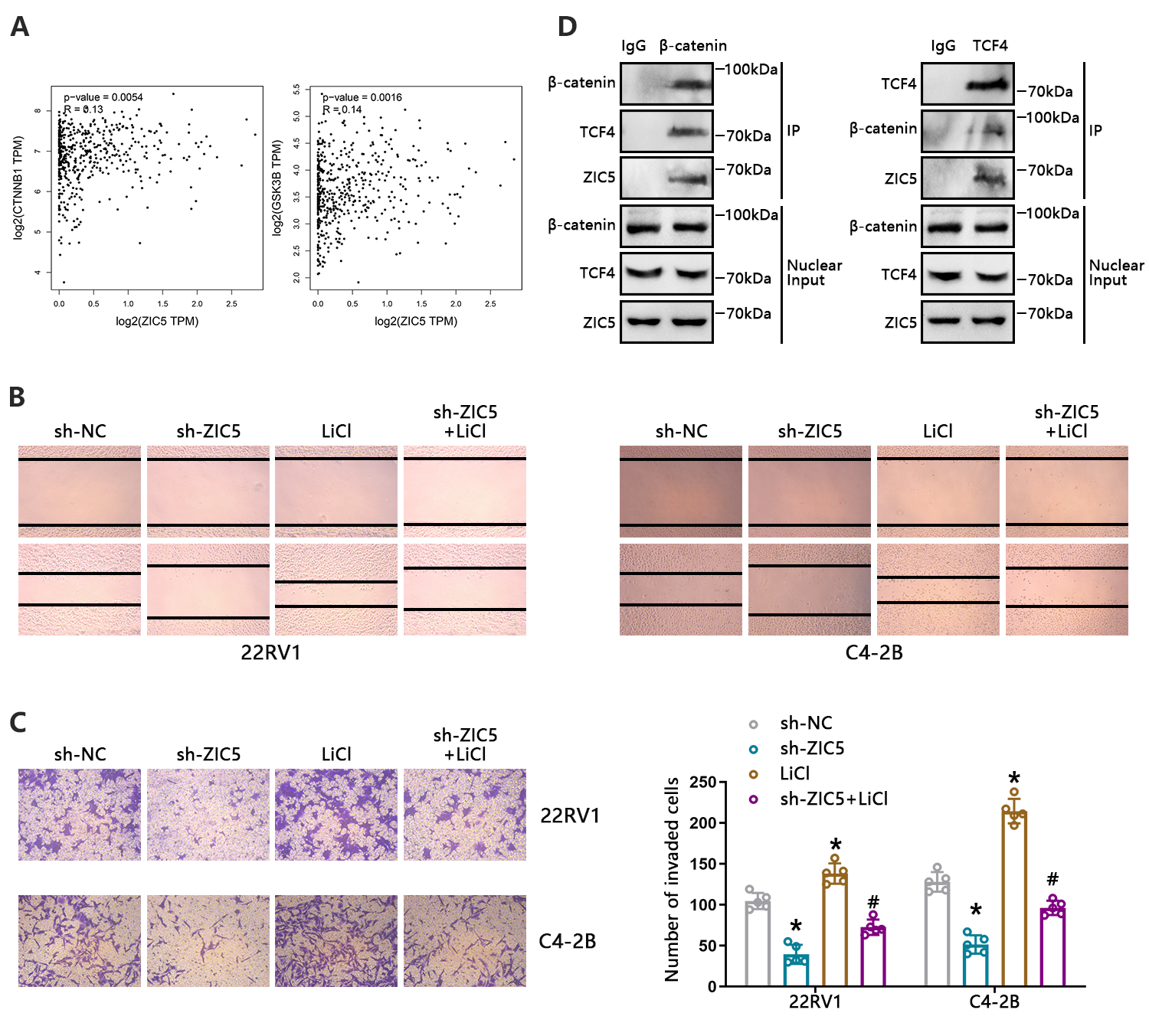

Supplement: Supplementary file 3 — Supplementary figure 3 [file 41420_2022_1181_MOESM3_ESM.tif]

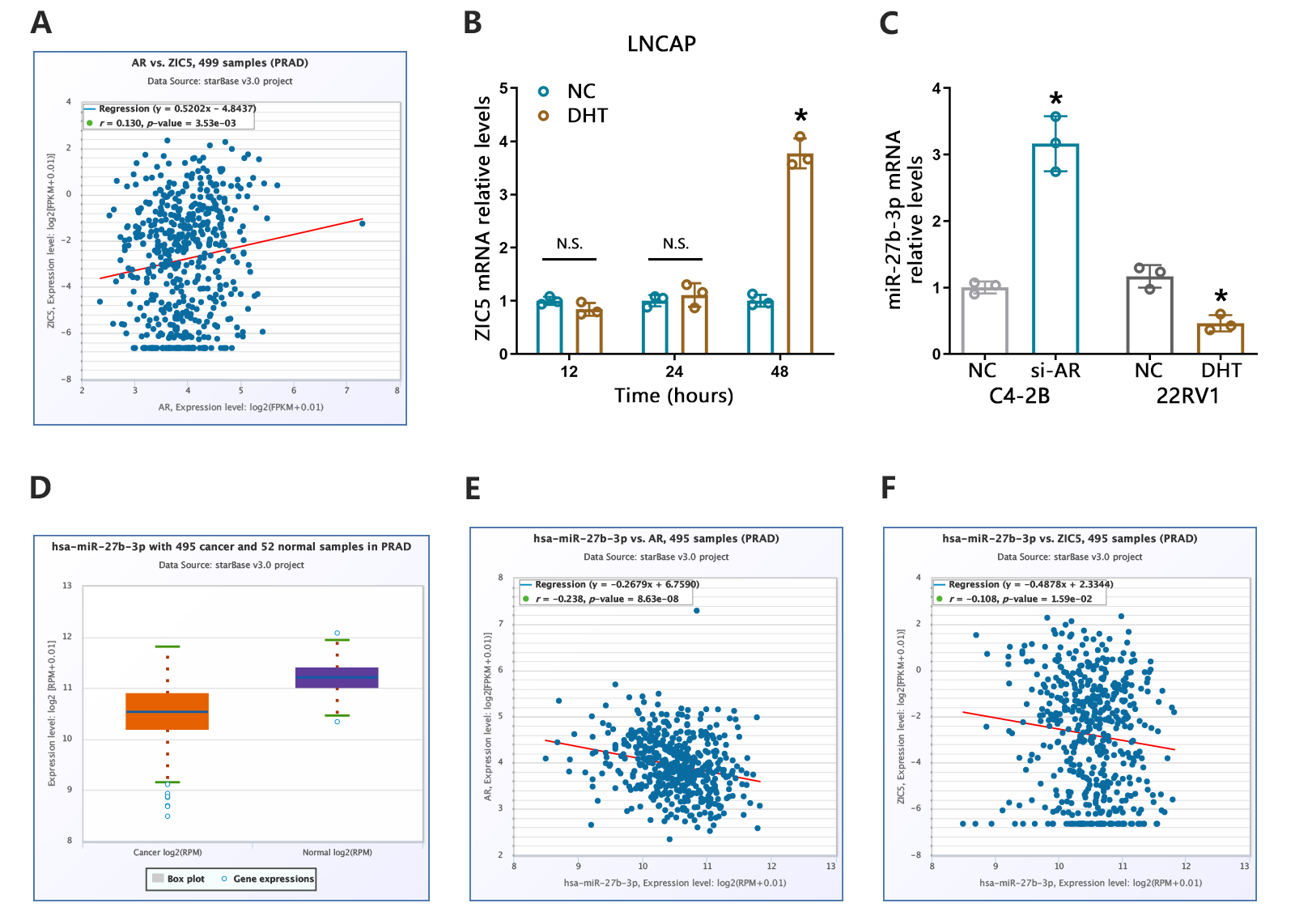

Supplement: Supplementary file 4 — Supplementary figure 4 [file 41420_2022_1181_MOESM4_ESM.tif]

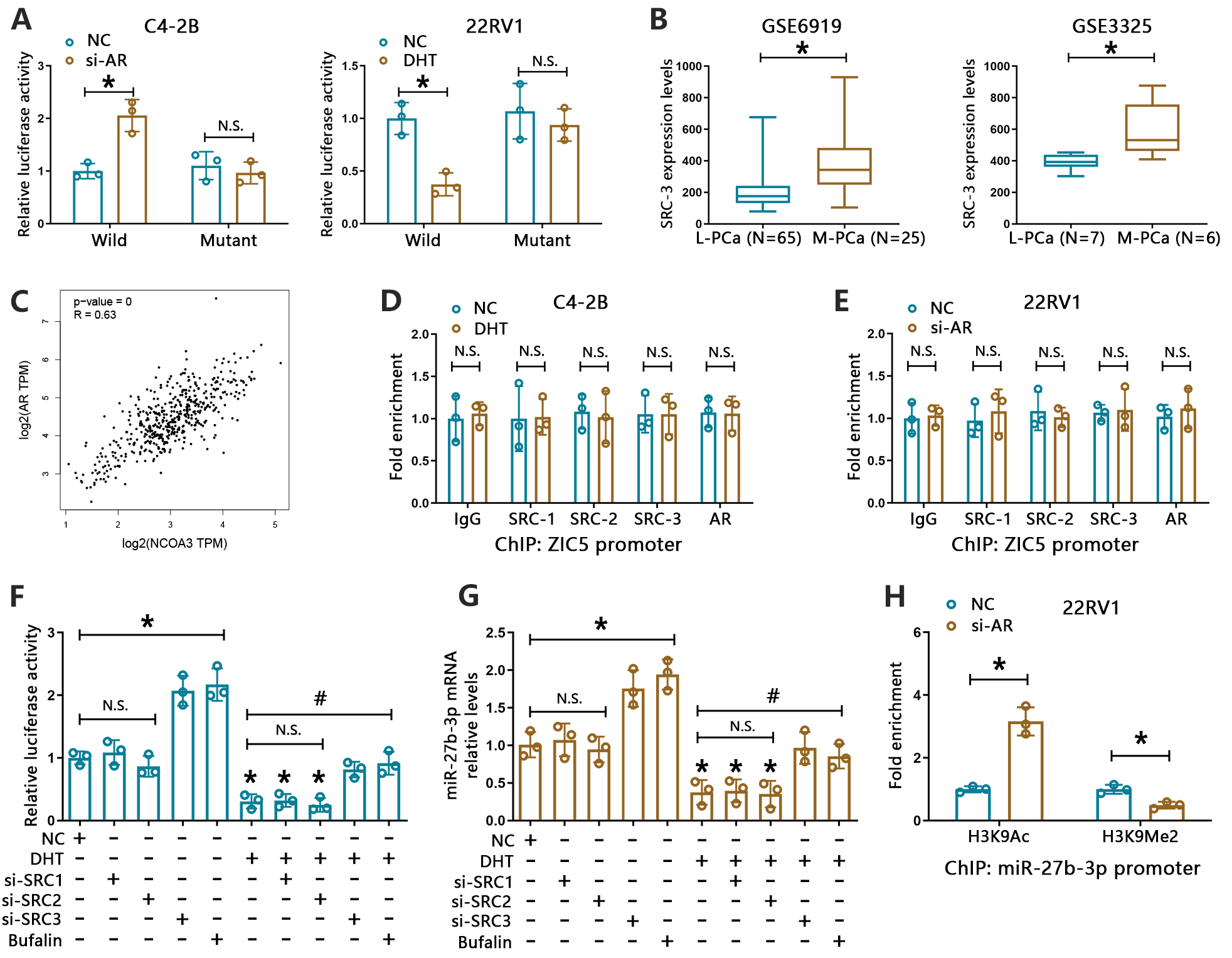

Supplement: Supplementary file 5 — Supplementary figure 5 [file 41420_2022_1181_MOESM5_ESM.tif]

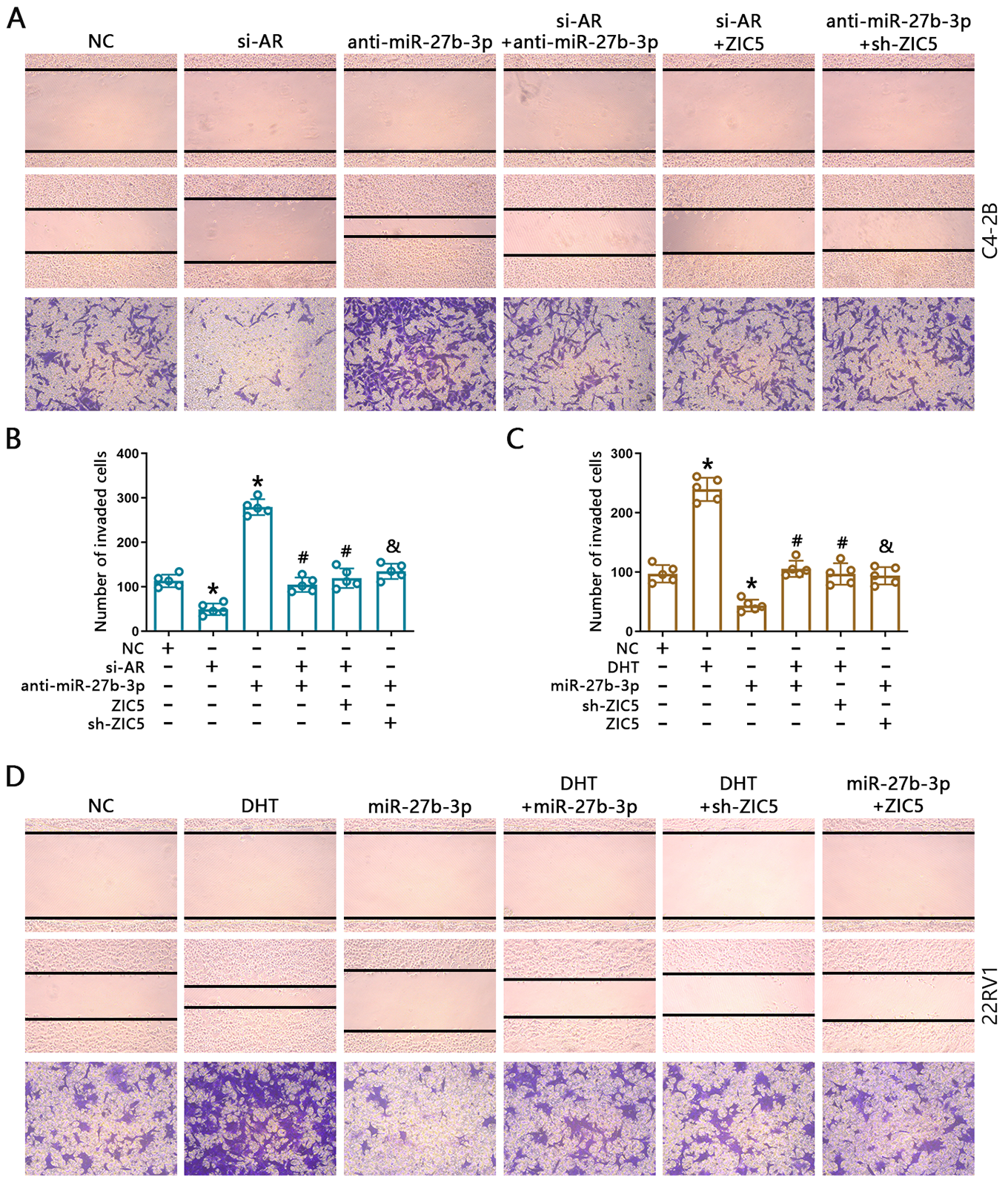

Supplement: Supplementary file 6 — Supplementary figure 6 [file 41420_2022_1181_MOESM6_ESM.tif]

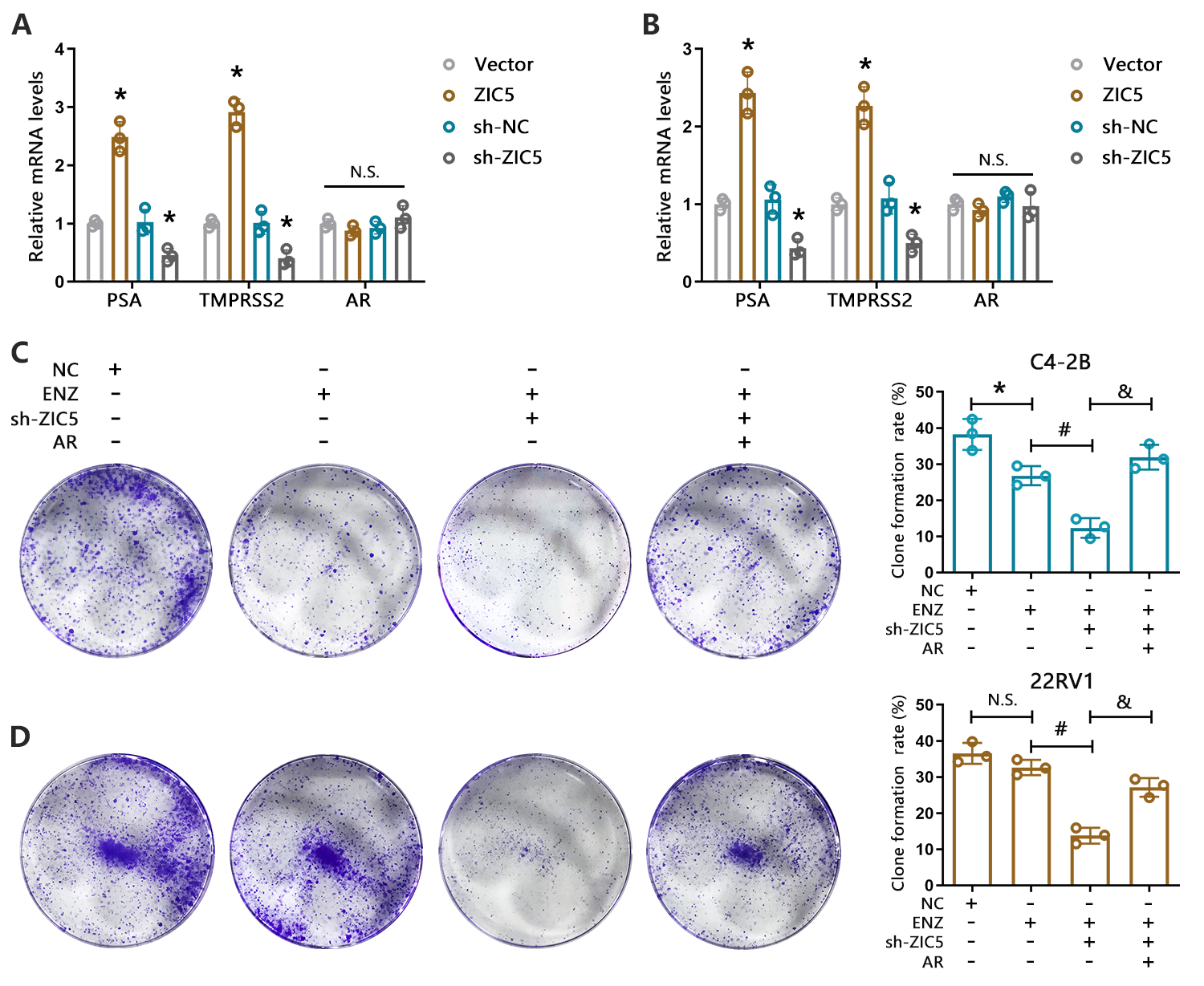

Supplement: Supplementary file 7 — Supplementary figure 7 [file 41420_2022_1181_MOESM7_ESM.tif]

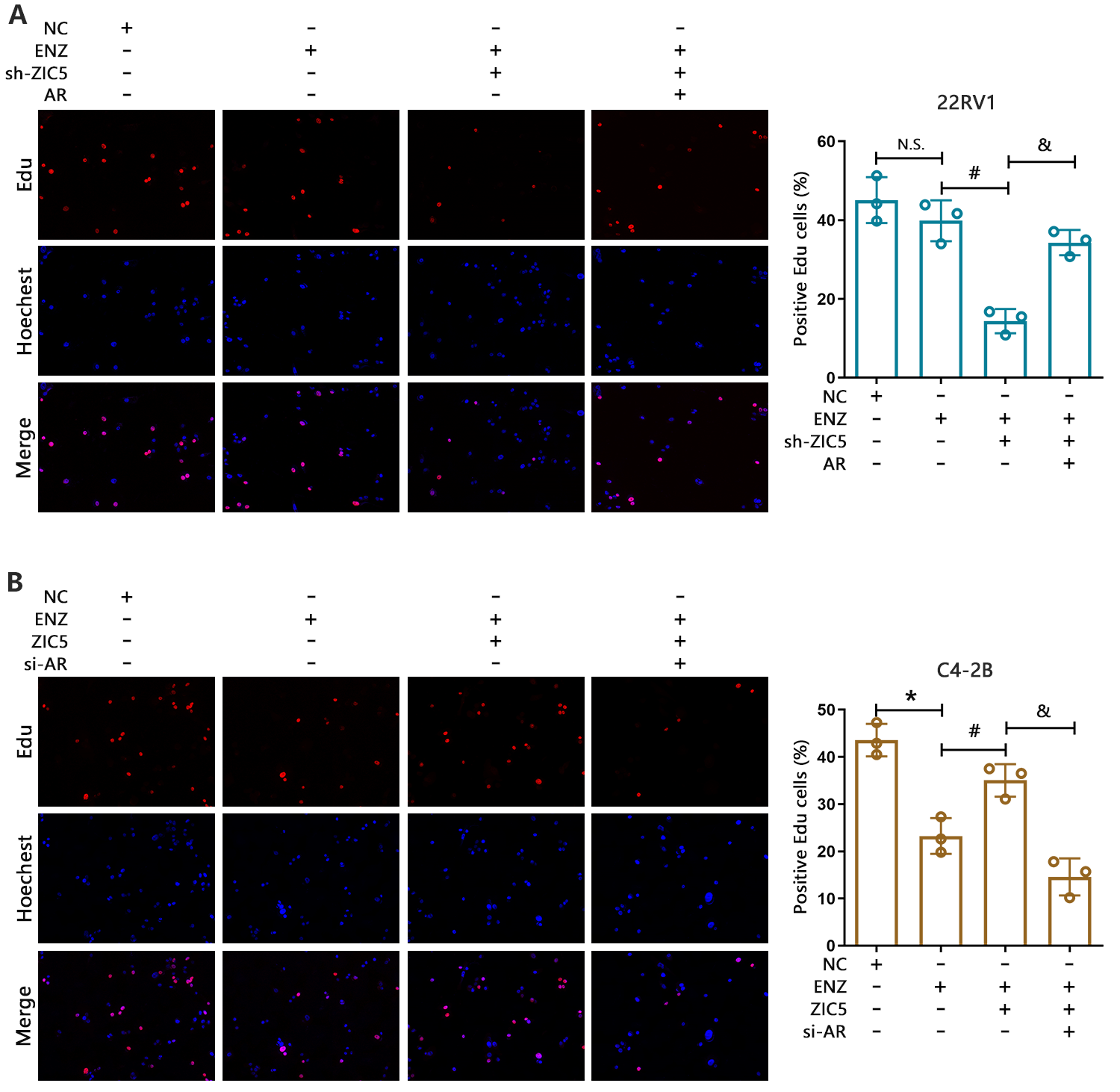

Supplement: Supplementary file 8 — Supplementary figure 8 [file 41420_2022_1181_MOESM8_ESM.tif]

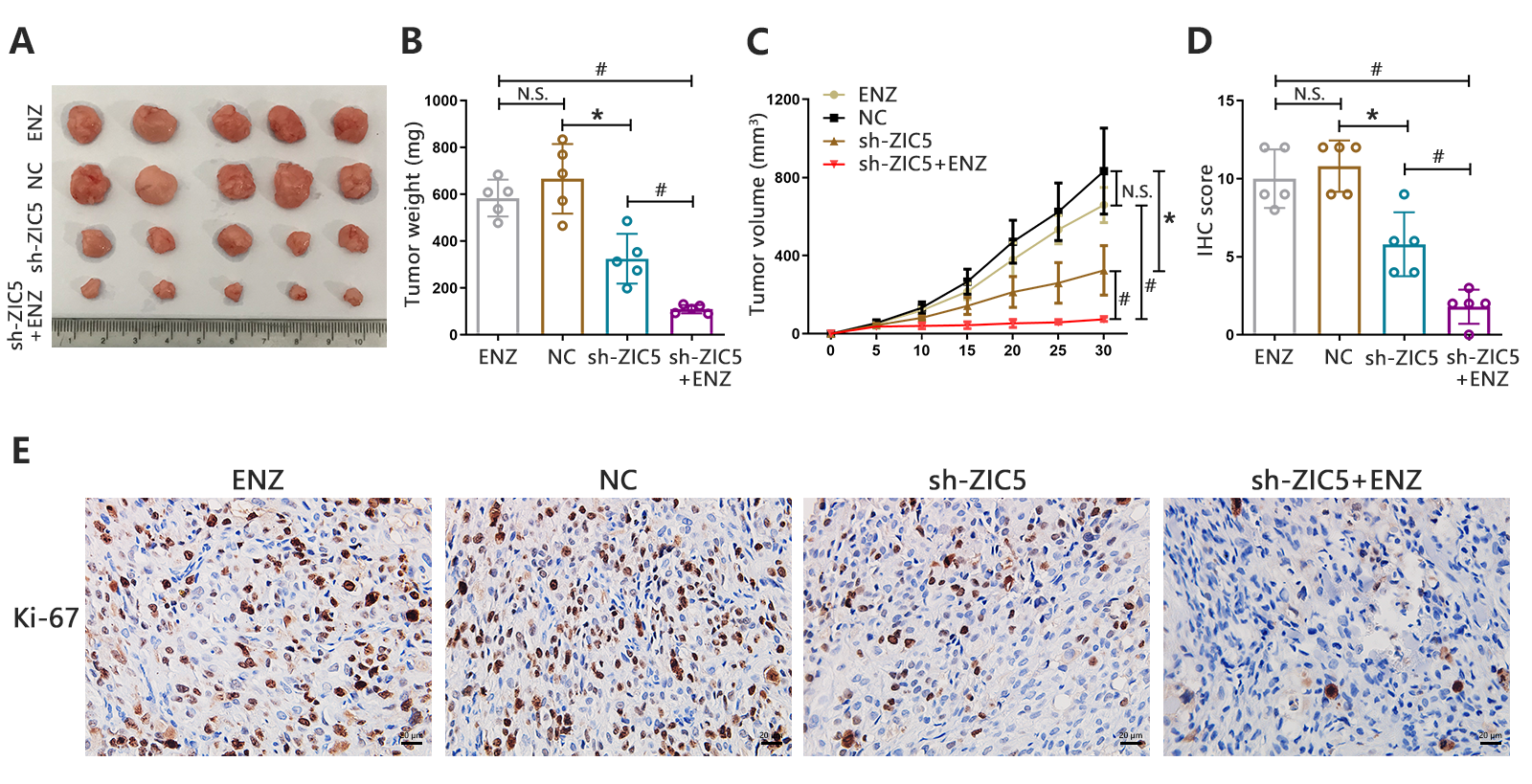

Supplement: Supplementary file 9 — Supplementary figure 9 [file 41420_2022_1181_MOESM9_ESM.tif]
