# Supplementary material for: AR-regulated ZIC5 contributes to the aggressiveness of prostate cancer
Source: Cell Death Discov. 2022 Sep 20;8:393. doi: 10.1038/s41420-022-01181-4 (PMC9489711; doi:10.1038/s41420-022-01181-4)
Supplement: Supplementary file 10 — Supplementary Figure legends [file 41420_2022_1181_MOESM10_ESM.doc]

**Supplementary Fig. 1. Expression of ZIC5 in PCa cells and tissues. (A, B)** C4-2B (A) and 22RV1 (B) cells were transfected with ZIC5-targeted shRNA (sh-ZIC5) or overexpression plasmid (oe-ZIC5), and western blotting and RT-qPCR assays were carried out to detect transfection efficiency. *P<0.05, relative to NC group. **(C)** Clinical expression of ZIC5 in localized and metastatic PCa (GEO dataset GSE3325). **(D**, **E)** Representative images of colony formation assays conducted in C4-2B and 22RV1 cells following knockdown or overexpression of ZIC5. *P<0.05 vs. NC group.

**(F, G)** Results of wound healing and Transwell assays evaluating migratory and invasive abilities of PC3 cells transfected with sh-ZIC5 or oe-ZIC5. The number of invasive cells was counted in five random fields under 200X magnification. *P < 0.05, compared with NC, N.S., P>0.05 vs. NC.

**Supplementary Fig. 2. ZIC5 potentiates the migration and invasion of PCa cells. (A**, **B)** Results of wound healing and Transwell assays evaluating migratory and invasive abilities of 22RV1 and C4-2B cells transfected with sh-ZIC5 or oe-ZIC5. The number of invasive cells was counted in five random fields under 200X magnification. *P < 0.05, compared with sh-NC or oe-NC. **(C**, **D)** 22RV1 and C4-2B cells were cotransfected with oe-ZIC5 and TWIST-targeted siRNA. Cells’ migratory and invasive abilities were evaluated by wound healing and Transwell assays. The number of invasive cells was counted in five random fields under 200X magnification. *P < 0.05, compared with oe-NC, N.S., P>0.05 vs. NC, ^#^P < 0.05, compared with oe-ZIC5.

**Supplementary Fig. 3. ZIC5 enhances PCa cell migration and invasion via Wnt/β-catenin signaling. (A)** Analysis of the correlation between ZIC5, CTNNB1 (β-catenin), and GSK3B expression in TCGA-PCa patients (GEPIA platform). **(B**, **C)** Analysis of cell migration (B) and invasion (C) in 22RV1 and C4-2B cells transfected with sh-NC or sh-ZIC5 and treated with LiCl. Invasive cells were counted in five random fields under 200X magnification. *P < 0.05, compared with sh-NC, ^#^P<0.05 vs. sh-ZIC5. **(D)** Co-IP assay results showing interaction between endogenous β-catenin and ZIC5, and endogenous TCF4 and ZIC5 in 293T cells.

**Supplementary Fig. 4. AR induces ZIC5 expression via downregulation of miR-27b-3p. (A)** Analysis of the association between AR and ZIC5 expression in PCa patients from TCGA (ENCORI platform). **(B)** RT-qPCR determination of relative ZIC5 mRNA levels in LNCAP cells treated with DHT (1 nmol/L) for 12, 24, or 48 h. *P<0.05 vs. NC group, N.S. P>0.05 vs. NC. **(C)** RT-qPCR detection of relative miR-27b-3p levels in 22RV1 cells treated with DHT (1 nmol/L) and in C4-2B cells transfected with AR-specific siRNA. *P < 0.05, compared with NC. **(D)** Expression of miR-27b-3p in human normal prostate and PCa samples (TCGA data in ENCORI). **(E**, **F)** Analysis of the correlation between miR-27b-3p, AR, and ZIC5 expression levels in PCa patients (TCGA data in ENCORI).

**Supplementary Fig. 5. AR association with SRC-3 modulates the transcription of miR-27b-3p. (A)** Analysis of AR/miR-27b-3p promoter interaction in C4-2B cells cotransfected with si-AR and miR-27b-3p promoter luciferase reporter or mutant vector, or in DHT-treated 22RV1 cells transfected with miR-27b-3p promoter luciferase reporter or mutant vector. *P<0.05, relative to NC, N.S. P>0.05 vs. NC.  **(B)** Expression of ZIC5 in localized tumor and metastatic PCa patient samples (GEO dataset GSE6919 and GSE3325). *P<0.05 vs. localized PCa tumors. **(C)** Analysis of the association between AR and SRC-3 (NCOA3) expression in PCa patients from TCGA (GEPIA platform). **(D)** ChIP-qPCR analysis to assess specific binding of SRC-1, SRC-2, SRC-3 and AR to the ZIC5 promoter in C4-2B cells treated with or without DHT. Purified IgG was used as control. N.S. P>0.05 vs. NC. **(E)** ChIP-qPCR analysis to assess specific binding of SRC-1, SRC-2, SRC-3 and AR to the ZIC5 promoter in 22RV1 cells transfected with control or AR-targeted siRNA. Purified IgG was used as control. N.S., P>0.05 vs. NC. **(F)** Luciferase-based detection of miR-27b-3p promoter activity in 22RV1 cells exposed to the indicated treatments. *P<0.05 vs. NC, ^#^P<0.05 vs. DHT, N.S. P>0.05 vs. NC or DHT. **(G)** RT-qPCR analysis of relative miR-27b-3p levels in 22RV1 cells treated with or without DHT following administration of bufalin (50nM, 24 h) or indicated SRC-targeted siRNA (48 h). *P<0.05 vs. NC, ^#^P<0.05 vs. DHT, N.S. P>0.05 vs. NC or DHT. **(H)** ChIP-qPCR analysis to assess specific binding of H3K9Ac and H3K9Me2 to the miR-27b-3p promoter in 22RV1 cells transfected with control or AR-targeted siRNA. Purified IgG was used as control. *P<0.05 vs. NC.

**Supplementary Fig. 6. AR-mediated miR-27b-3p downregulation contributes to PCa cell migration and invasion by enhancing ZIC5 protein expression. (A)** Wound healing and Transwell assays were employed for analysis of migration and invasion capacities of C4-2B cells following experimental modulation of AR, miR-27b-3p, and ZIC5 expression. **(B)** Quantification of invasive C4-2B cells in five random fields under 200X magnification. *P<0.05 vs. NC, ^#^P<0.05 vs. si-AR, ^&^P<0.05 vs. anti-miR-27b-3p (miR-27b-3p inhibitors). **(C)** Quantification of invasive 22RV1 cells in five random fields under 200X magnification. *P<0.05 vs. NC, ^#^P<0.05 vs. DHT, ^&^P<0.05 vs. miR-27b-3p (miR-27b-3p mimics). **(D)** Analysis of migration and invasion capacities of 22RV1 cells following experimental modulation of AR, miR-27b-3p, and ZIC5 expression.

**Supplementary Fig. 7. ZIC5 promotes enzalutamide resistance through AR. (A**, **B)** RT-qPCR analysis of AR and AR target genes (PSA, TMPRSS2) in LNCAP (A) and C4-2B (B) cells after transfection sh-ZIC5 or oe-ZIC5 for 48 h. *P<0.05 vs. control vector or sh-NC, N.S. P>0.05 vs. control vector. **(C**, **D)** Colony formation assay results. C4-2B and 22RV1 cells were transfected as indicated with ZIC5- or AR-specific shRNAs or expression plasmids, and treated with ENZ (20 μmol/L) for 72 h. N.S., P>0.05 vs. NC, *P<0.05 vs. NC, ^#^P<0.05 vs. ENZ, ^&^P<0.05 vs. ENZ + sh-ZIC5.

**Supplementary Fig. 8. ZIC5 inhibition boosts the antiproliferative effect of enzalutamide on PCa cells. (A**, **B)** 22RV1 and C4-2B cells were treated with ENZ (20 μmol/L) for 72 h after transfection with the indicated vectors or plasmids. EdU staining was applied for analysis of cell proliferation. The numbers of EdU- and Hoechst-positive cells were counted in three random fields. Scale bars, 50 μm, N.S., P>0.05 vs. NC, *P < 0.05 vs. NC, ^#^P<0.05 vs. ENZ, ^&^P<0.05 vs. ENZ + sh-ZIC5 or ENZ + ZIC5.

**Supplementary Fig. 9. ZIC5 inhibition increases the efficacy of enzalutamide in mice.** 22RV1 cells stably expressing ZIC5-targeted shRNA or NC shRNA were implanted subcutaneously into the dorsal flanks of nude mice, followed by administration of enzalutamide or vehicle. **(A**, **B)** Representative images of excised tumors and tumor weight measurements. N.S., P>0.05 vs. NC, *P < 0.05 vs. sh-ZIC5, ^#^P<0.05 vs. sh-ZIC5+ENZ. **(C)** Tumor growth curve. N.S., P>0.05 vs. NC, *P < 0.05 vs. sh-ZIC5, ^#^P<0.05 vs. sh-ZIC5+ENZ. **(D**, **E)** IHC analysis of Ki-67 expression in xenografted PCa samples and corresponding IHC scores. N.S., P>0.05 vs. NC, *P<0.05 vs. sh-ZIC5, ^#^P<0.05 vs. sh-ZIC5+ENZ.
