# Supplementary material for: AR-regulated ZIC5 contributes to the aggressiveness of prostate cancer
Source: Cell Death Discov. 2022 Sep 20;8:393. doi: 10.1038/s41420-022-01181-4 (PMC9489711; doi:10.1038/s41420-022-01181-4)
Supplement: Supplementary file 11 — Supplementary Table 1 [file 41420_2022_1181_MOESM11_ESM.docx]

**Supplementary Table 1:** RT-qPCR primer sequences

| Gene name | Sequence (5′-3′) |
| --- | --- |
| ZIC5 | Forward: CAAGCGTACTCATACAGGGGAA |
|  | Reverse: GGAGCTTGGGTGAGTGTAGG |
| N-cadherin | Forward: GCTGGACCGAGAGAGTTTCC |
|  | Reverse: CGACGTTAGCCTCGTTCTCA |
| Snail1 | Forward: CGAGTGGTTCTTCTGCGCTA |
|  | Reverse: CTGCTGGAAGGTAAACTCTGGA |
| E-cadherin | Forward: GAGGCTTCTGGTGAAATCGC |
|  | Reverse: TGCAGTTGCTAAACTTCACATT |
| TWIST1 | Forward: GCCGGAGACCTAGATGTCATT |
|  | Reverse: TTTTAAAAGTGCGCCCCACG |
| MMP7 | Forward: AGTGGTCACCTACAGGATCG |
|  | Reverse: GGGATCTCTTTGCCCCACAT |
| MMP2 | Forward: ATGACAGCTGCACCACTGAG |
|  | Reverse: ATTTGTTGCCCAGGAAAGTG |
| c-Myc | Forward: CCGCTTCTCTGAAAGGCTCT |
|  | Reverse: CGGGAGGCTGCTGGTTTT |
| MiR-27b-3p | Forward: GCGCGTTCACAGTGGCTAAG |
|  | Reverse: AGTGCAGGGTCCGAGGTATT |
| AR | Forward: AAGCAGGGATGACTCTGGGA |
|  | Reverse: CTGGGTTGTCTCCTCAGTGG |
| PSA | Forward: GCCTGGATCTGAGAGAGATATCATC |
|  | Reverse: ACACCTTTTTTTTTCTGGATTGTTG |
| TMPRSS2 | Forward: ATCGACAAATGAGGACGGCT |
|  | Reverse: AATCATGCACGGGGAAGCAA |
| AR-V7 | Forward: GAAGCTGCAAGGTCTTCTTCAA |
|  | Reverse: GGTCTGGTCATTTTGAGATGC |
| GAPDH | Forward: GGAGCGAGATCCCTCCAAAAT |
|  | Reverse: GGCTGTTGTCATACTTCTCATGG |
