# Supplementary material for: AR-regulated ZIC5 contributes to the aggressiveness of prostate cancer
Source: Cell Death Discov. 2022 Sep 20;8:393. doi: 10.1038/s41420-022-01181-4 (PMC9489711; doi:10.1038/s41420-022-01181-4)

Figure 1E

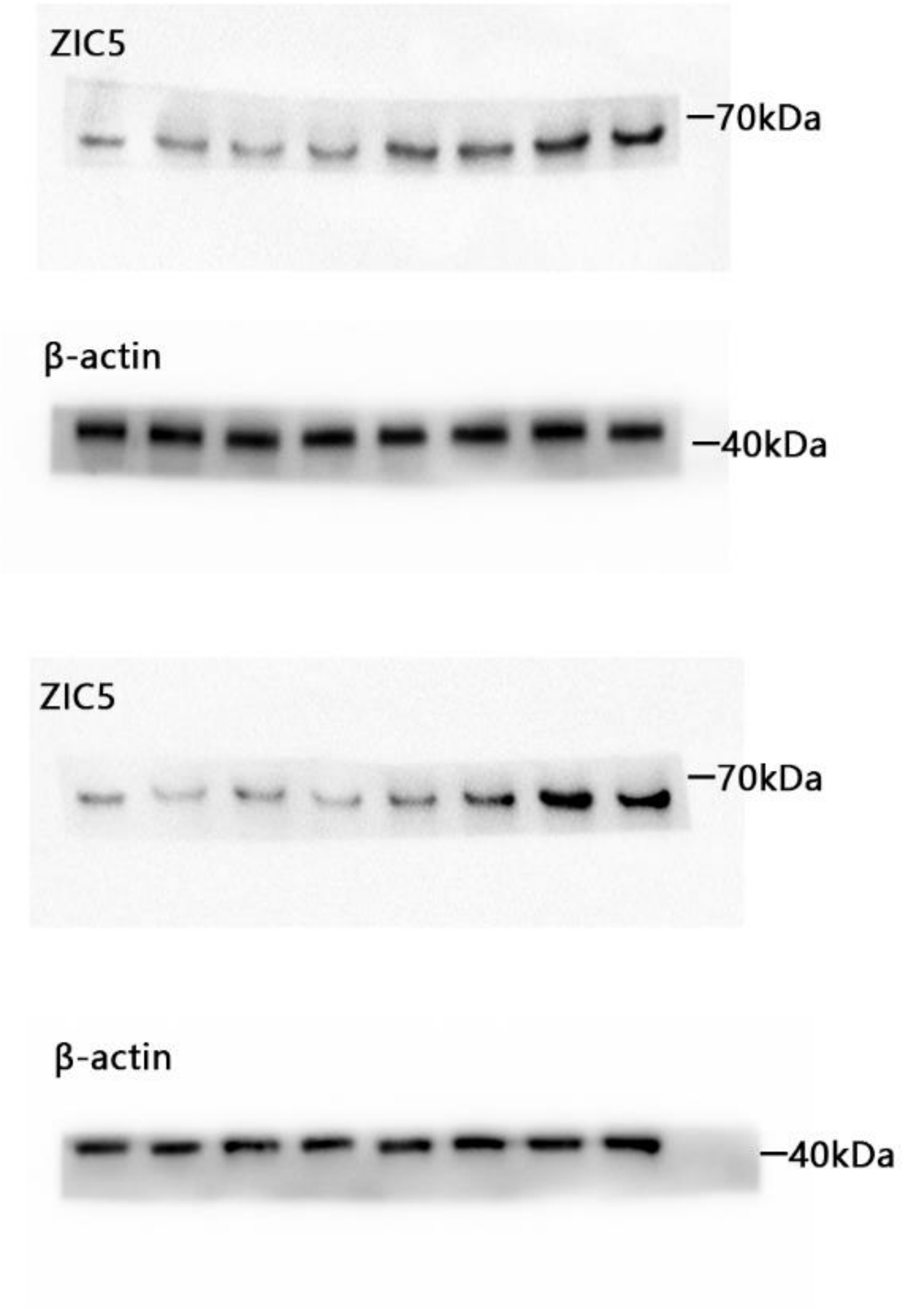

Figure 1G

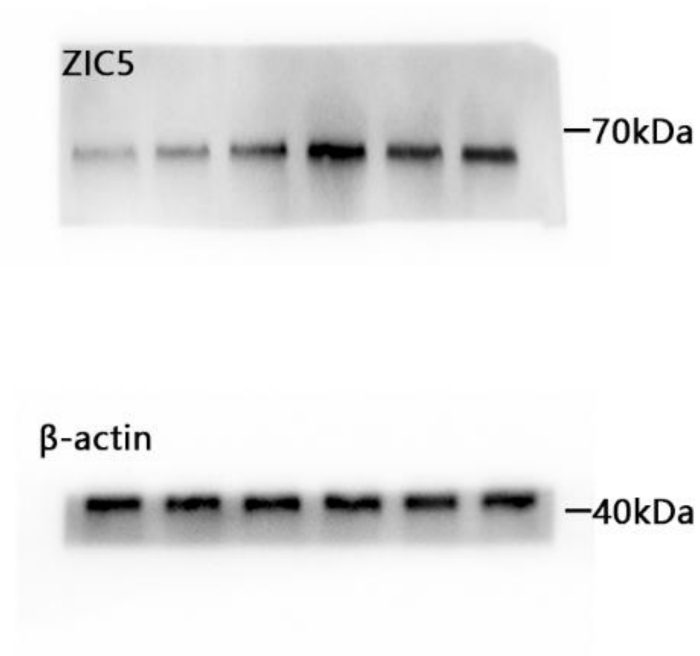

Figure 2B

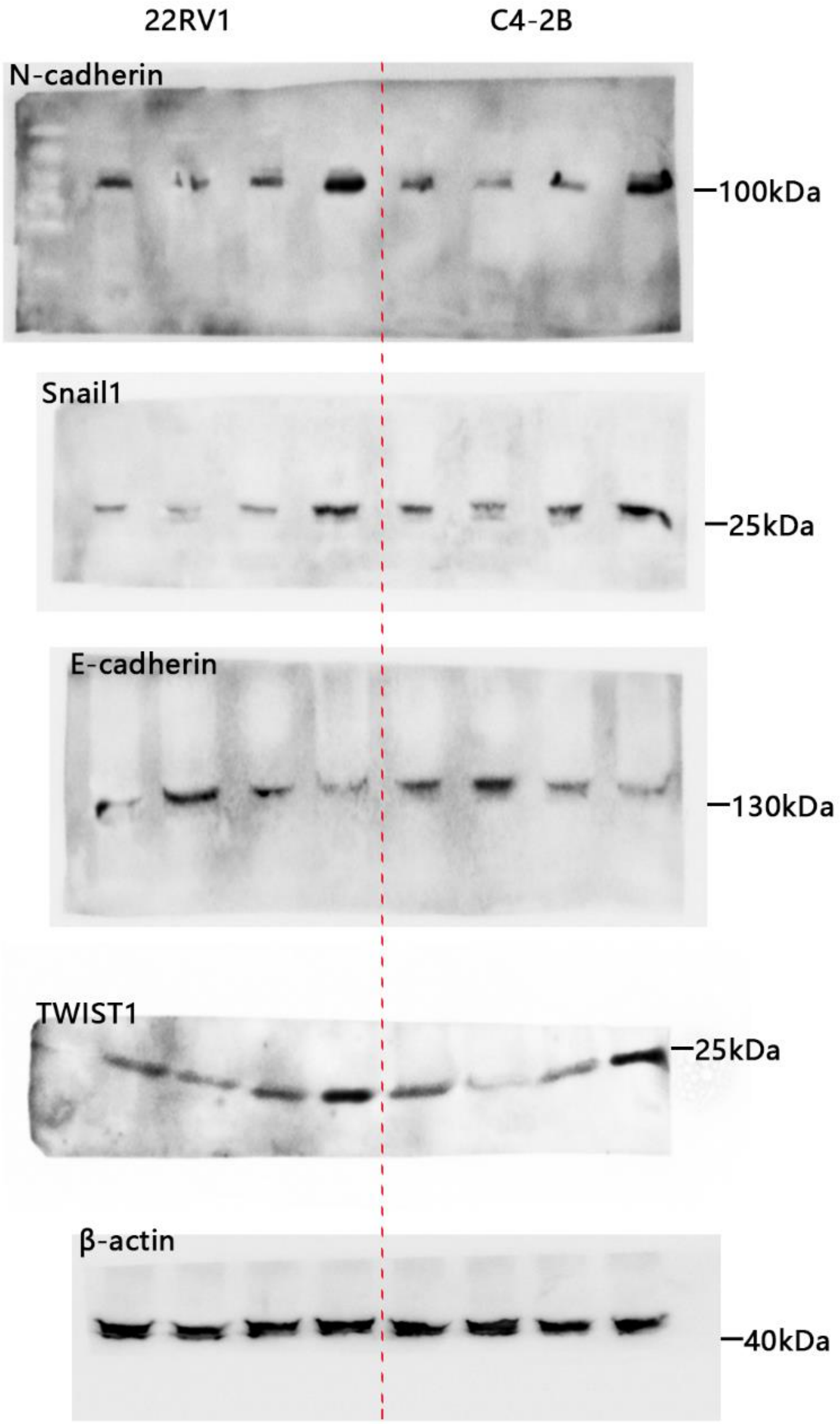
22RV1
C4-2B
N-cadherin
100kDa
Snail1
25kDa
E-cadherin
130kDa
TWIST1
25kDa
β-actin
40kDa

Figure 3F

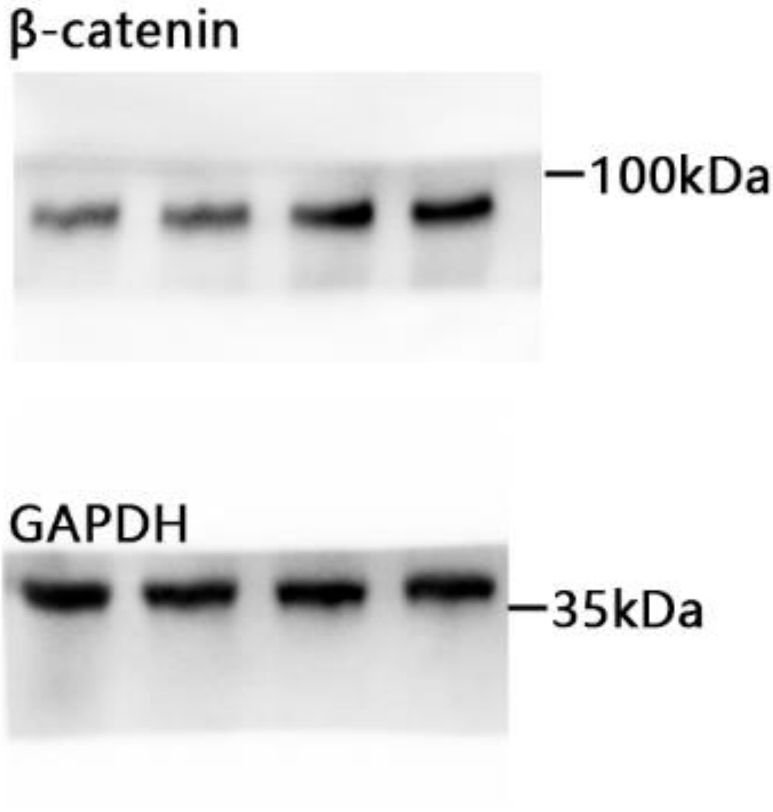

Figure 3G

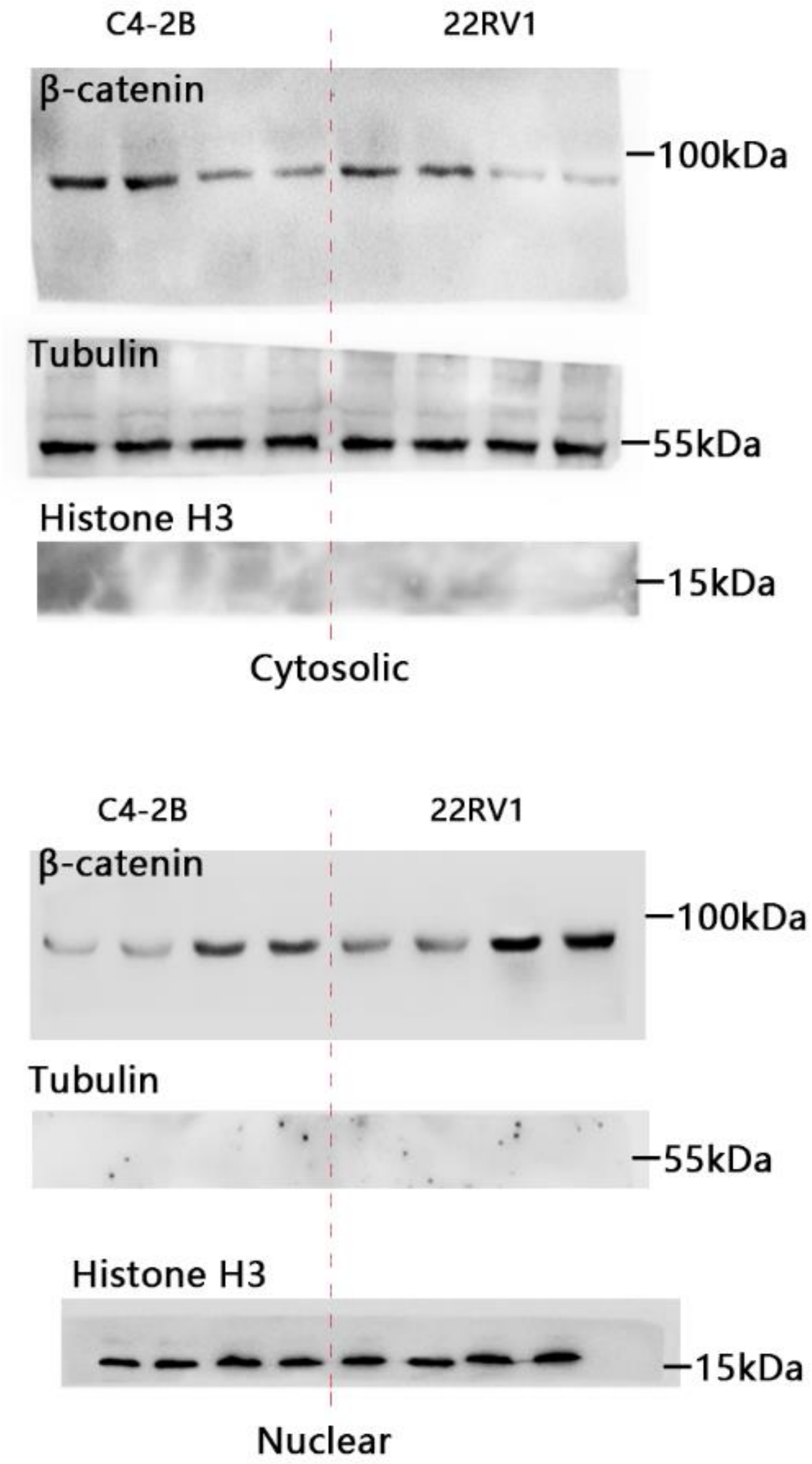
C4-2B
22RV1
β-catenin
100kDa
Tubulin
55kDa
Histone H3
15kDa
Cytosolic
C4-2B
22RV1
β-catenin
100kDa
Tubulin
55kDa
Histone H3
15kDa
Nuclear

Figure 3I

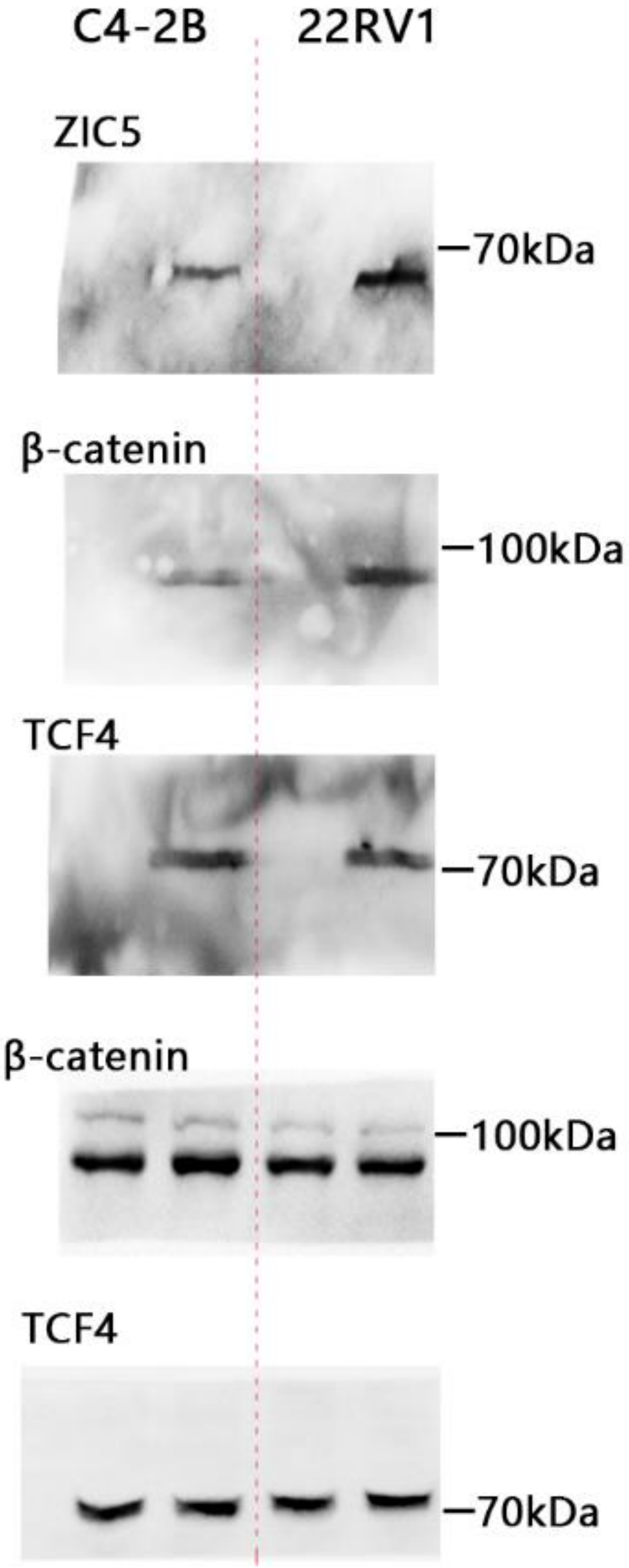
C4-2B
22RV1
ZIC5
70kDa
β-catenin
100kDa
TCF4
70kDa
β-catenin
100kDa
TCF4
70kDa

Figure 3J

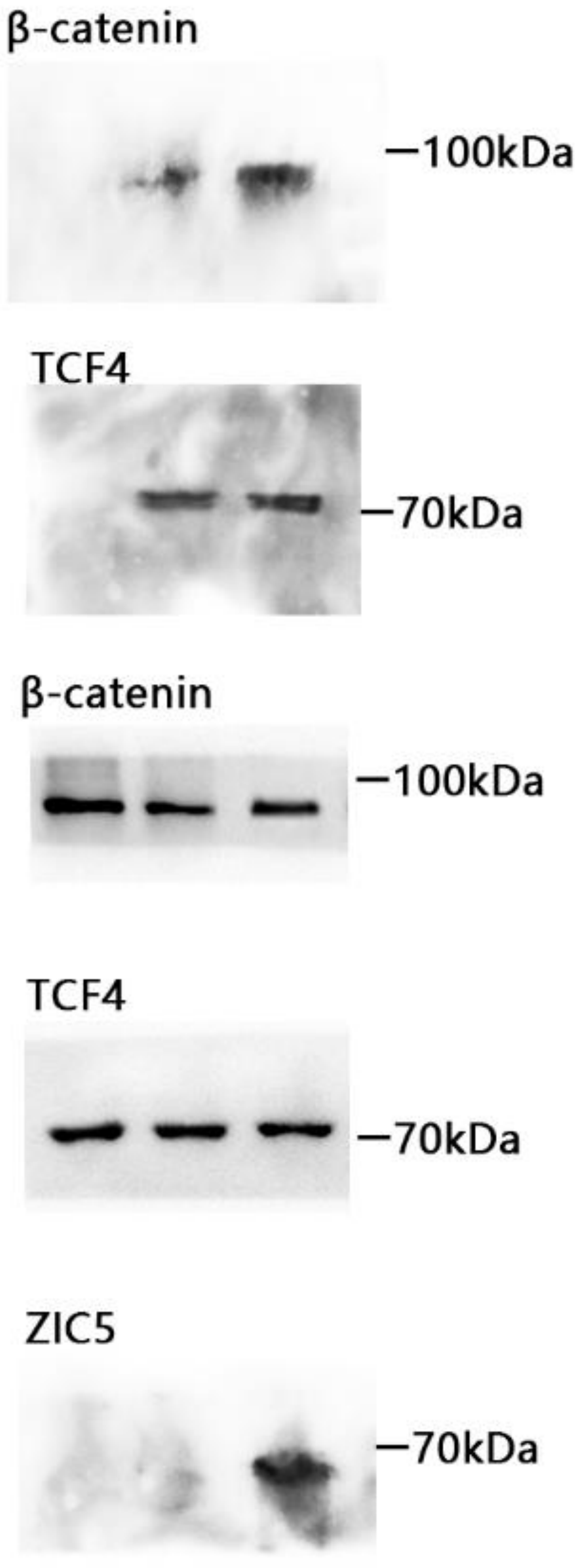
β-catenin
—100kDa
TCF4
—70kDa
β-catenin
—100kDa
TCF4
—70kDa
ZIC5
—70kDa

Figure 3K

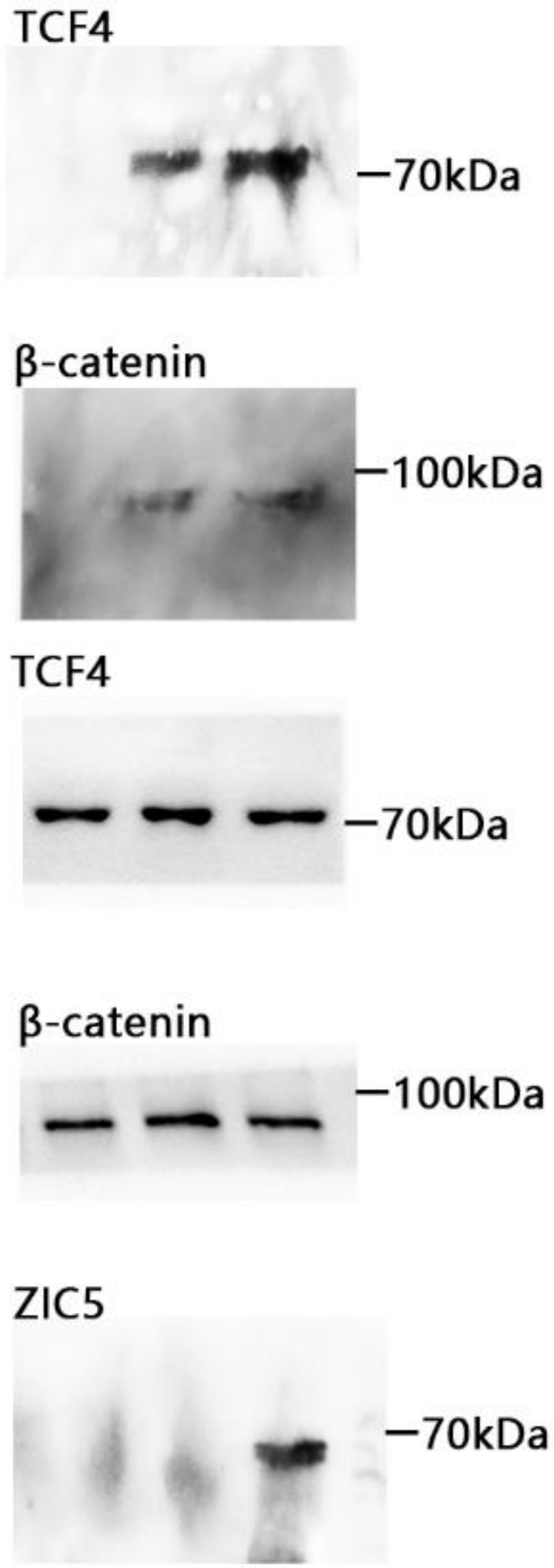
TCF4
70kDa
β-catenin
100kDa
TCF4
70kDa
β-catenin
100kDa
ZIC5
70kDa

Figure 4A

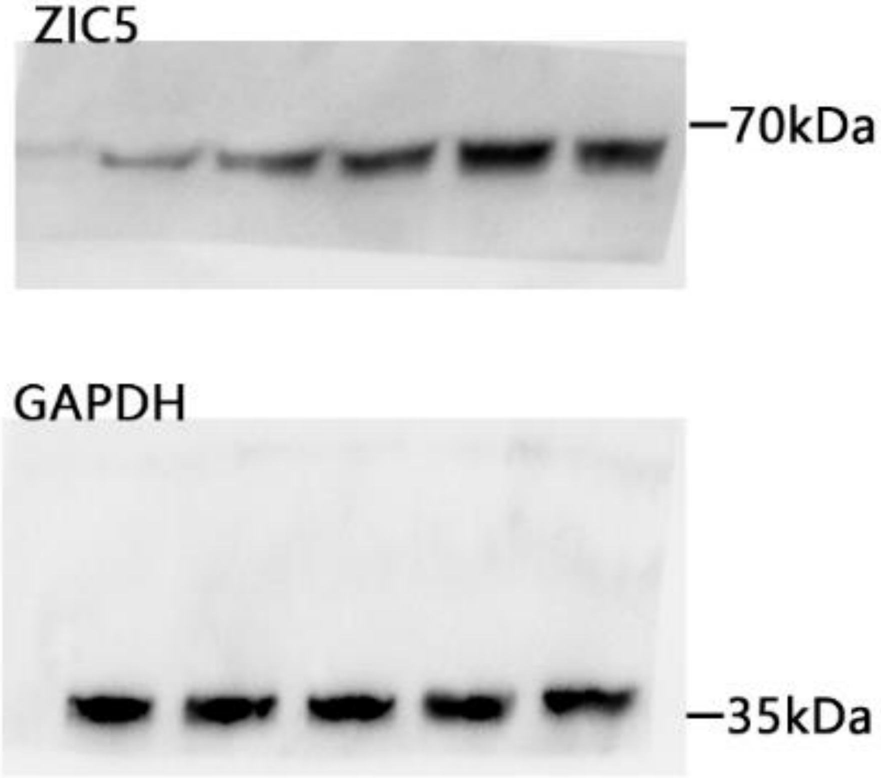

Figure 4B

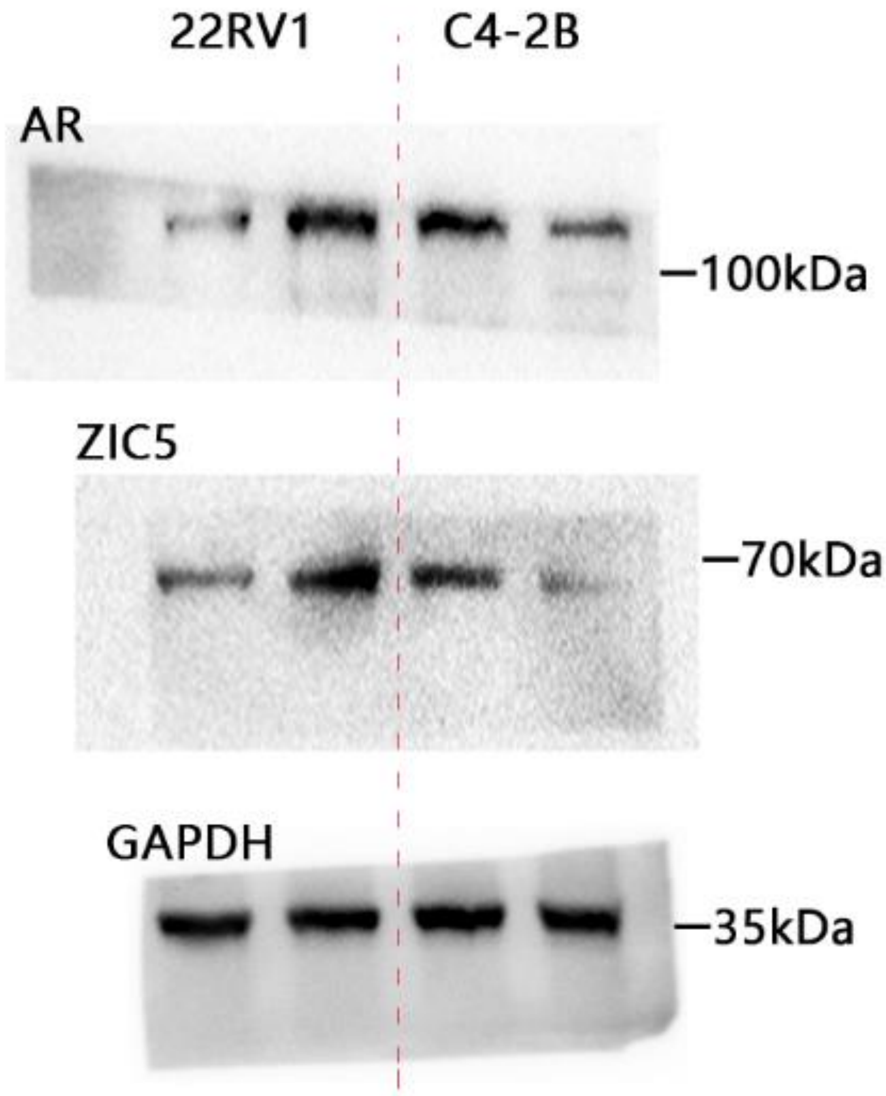

Figure 4C

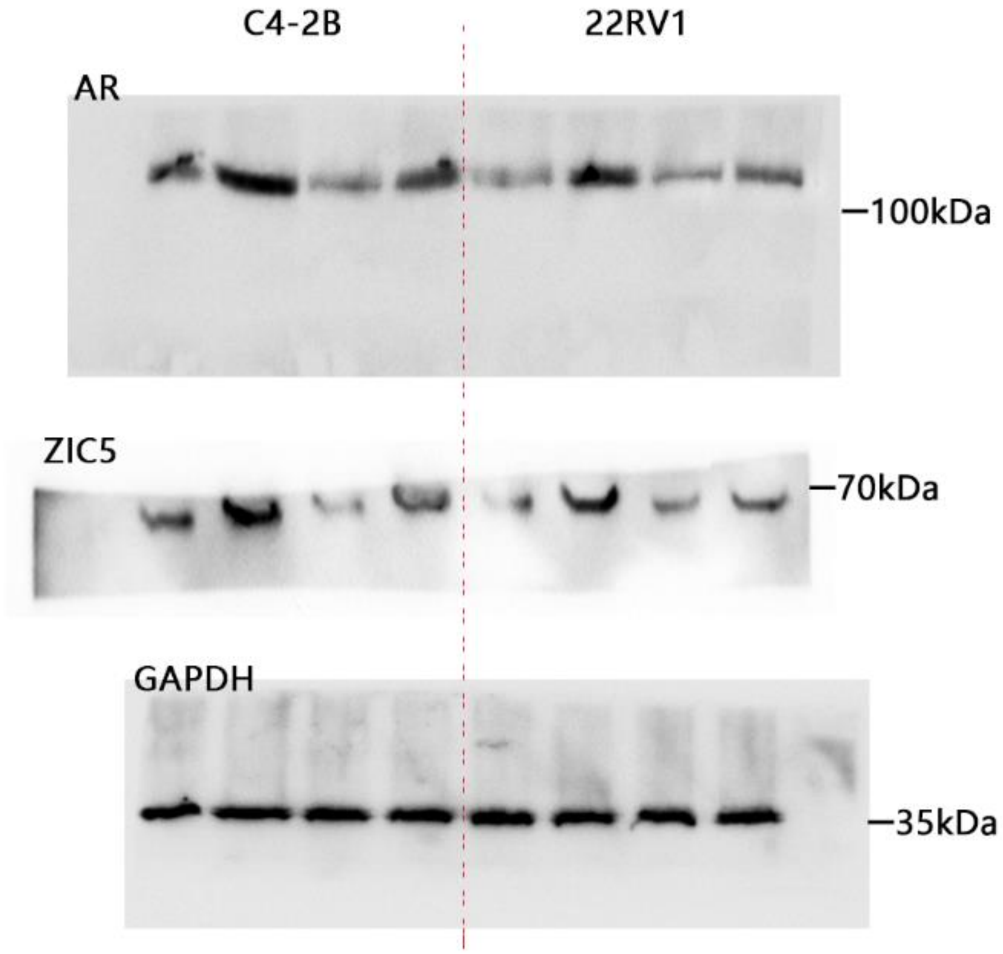

Figure 4G

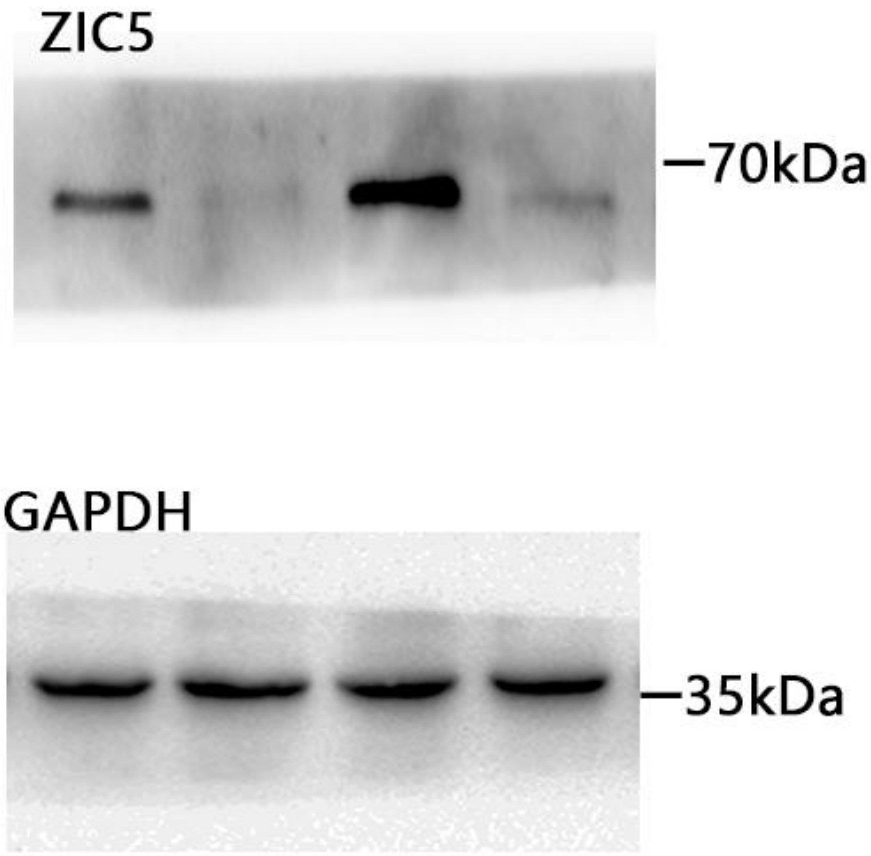

Figure 4K

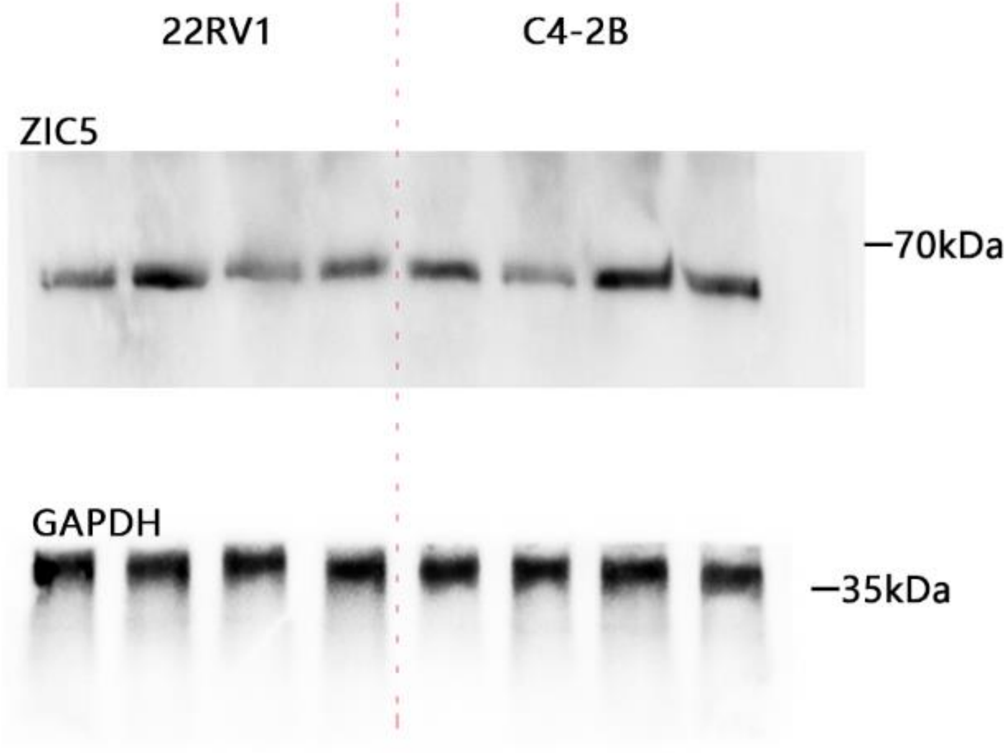

Figure 6B

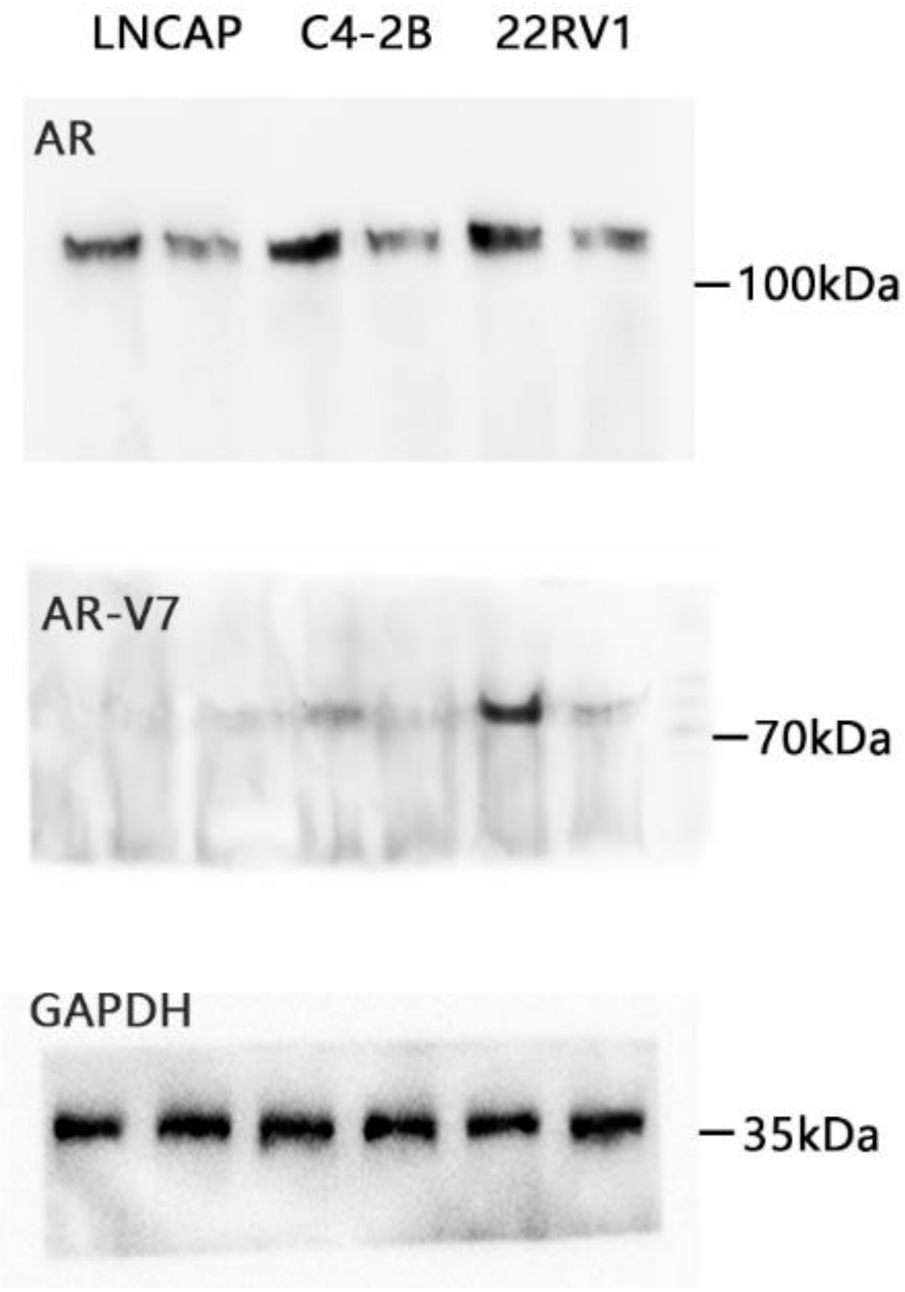

Supplementary figure 1A

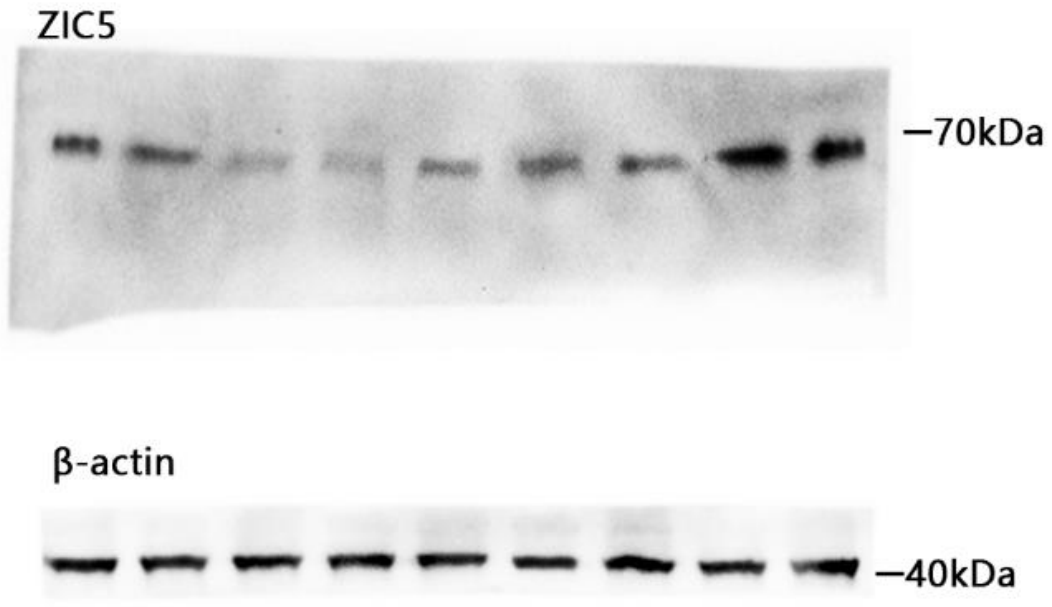

Supplementary figure 1B

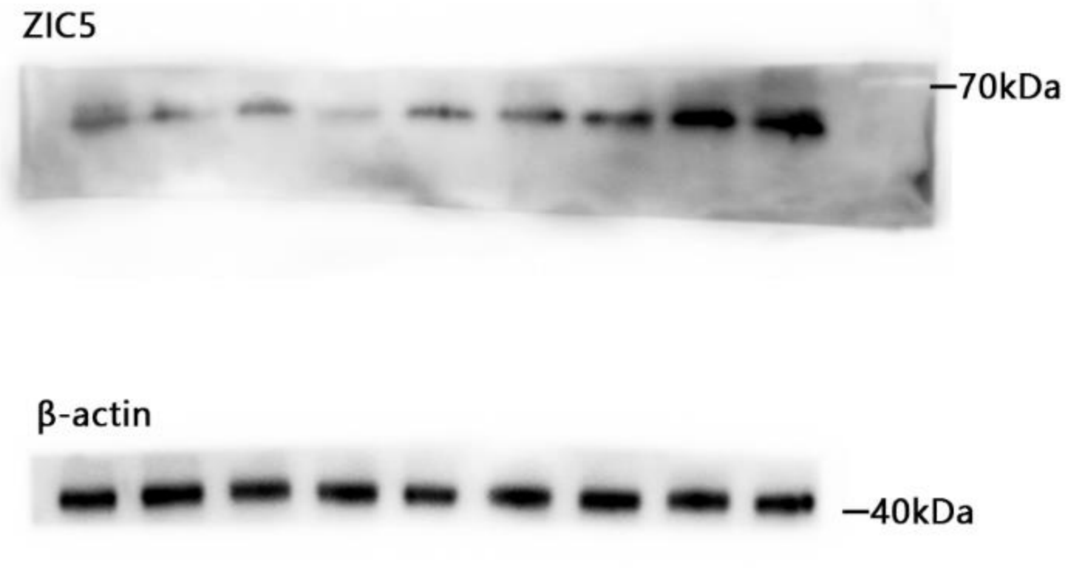

Supplementary figure 3D

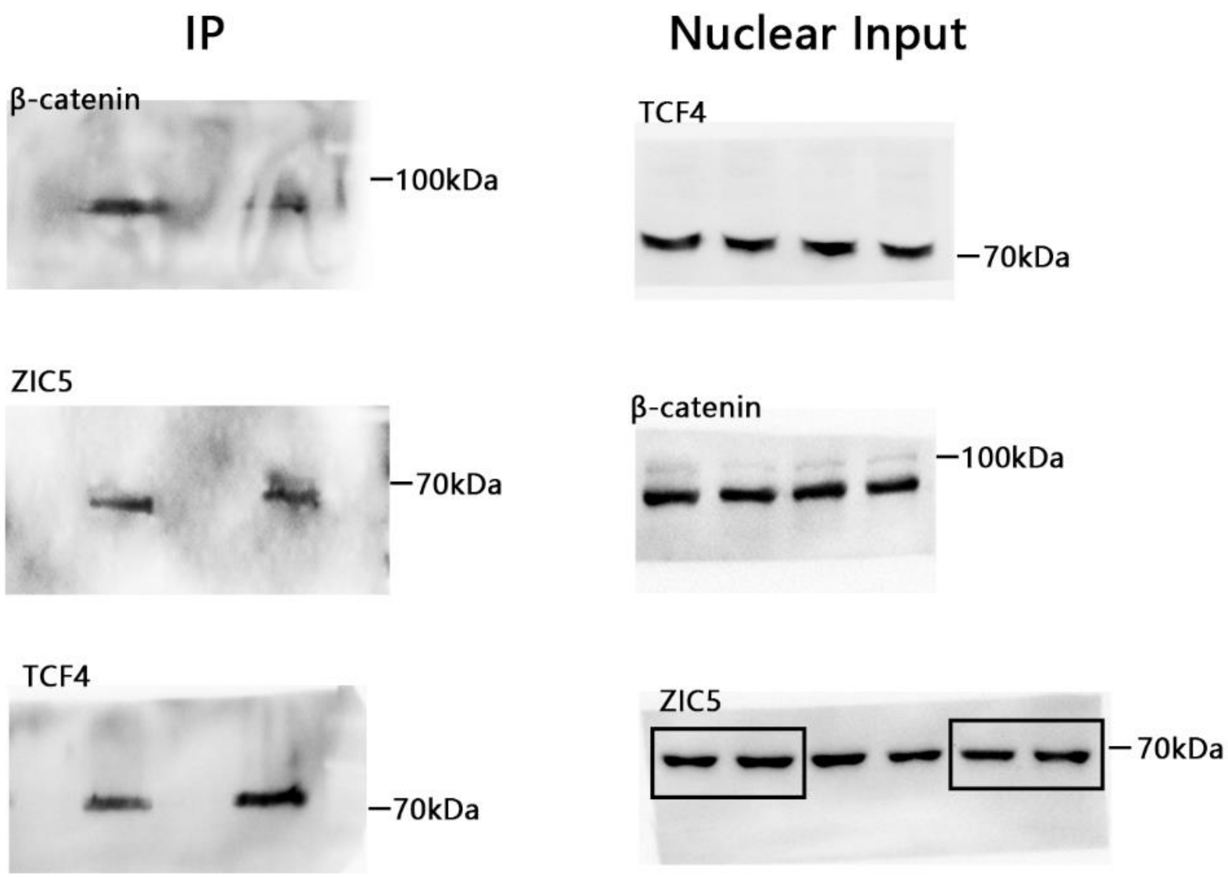

Supplement: Supplementary file 12 — Western blot original data [file 41420_2022_1181_MOESM12_ESM.pdf]
